# Rare subclonal sequencing of breast cancers indicates putative metastatic driver mutations are predominately acquired after dissemination

**DOI:** 10.1186/s13073-024-01293-9

**Published:** 2024-02-06

**Authors:** Matthew R. Lawrence-Paul, Tien-chi Pan, Dhruv K. Pant, Natalie N. C. Shih, Yan Chen, George K. Belka, Michael Feldman, Angela DeMichele, Lewis A. Chodosh

**Affiliations:** 1 2-PREVENT Translational Center of Excellence, Philadelphia, USA; 2 Abramson Family Cancer Research Institute, Philadelphia, USA; 3 Department of Cancer Biology, Perelman School of Medicine at the University of Pennsylvania, Philadelphia, PA 19104 USA; 4 Department of Pathology & Laboratory Medicine, Perelman School of Medicine at the University of Pennsylvania, Philadelphia, PA 19104 USA; 5 Department of Medicine, Perelman School of Medicine at the University of Pennsylvania, Philadelphia, PA 19104 USA; 6 Department of Biostatistics, Epidemiology and Informatics, Perelman School of Medicine at the University of Pennsylvania, Philadelphia, PA 19104 USA

**Keywords:** Breast cancer, Metastasis, Rare subclonal sequencing, Ultra-deep sequencing, Genomics, Metastatic driver mutation

## Abstract

**Background:**

Evolutionary models of breast cancer progression differ on the extent to which metastatic potential is pre-encoded within primary tumors. Although metastatic recurrences often harbor putative driver mutations that are not detected in their antecedent primary tumor using standard sequencing technologies, whether these mutations were acquired before or after dissemination remains unclear.

**Methods:**

To ascertain whether putative metastatic driver mutations initially deemed specific to the metastasis by whole exome sequencing were, in actuality, present within rare ancestral subclones of the primary tumors from which they arose, we employed error-controlled ultra-deep sequencing (UDS-UMI) coupled with FFPE artifact mitigation by uracil-DNA glycosylase (UDG) to assess the presence of 132 “metastasis-specific” mutations within antecedent primary tumors from 21 patients. Maximum mutation detection sensitivity was ~1% of primary tumor cells. A conceptual framework was developed to estimate relative likelihoods of alternative models of mutation acquisition.

**Results:**

The ancestral primary tumor subclone responsible for seeding the metastasis was identified in 29% of patients, implicating several putative drivers in metastatic seeding including *LRP5* A65V and *PEAK1* K140Q. Despite this, 93% of metastasis-specific mutations in putative metastatic driver genes remained undetected within primary tumors, as did 96% of metastasis-specific mutations in known breast cancer drivers, including *ERRB2* V777L, *ESR1* D538G, and *AKT1* D323H. Strikingly, even in those cases in which the rare ancestral subclone was identified, 87% of metastasis-specific putative driver mutations remained undetected. Modeling indicated that the sequential acquisition of multiple metastasis-specific driver or passenger mutations within the same rare subclonal lineage of the primary tumor was highly improbable.

**Conclusions:**

Our results strongly suggest that metastatic driver mutations are sequentially acquired and selected within the same clonal lineage both before, but more commonly after, dissemination from the primary tumor, and that these mutations are biologically consequential. Despite inherent limitations in sampling archival primary tumors, our findings indicate that tumor cells in most patients continue to undergo clinically relevant genomic evolution after their dissemination from the primary tumor. This provides further evidence that metastatic recurrence is a multi-step, mutation-driven process that extends beyond primary tumor dissemination and underscores the importance of longitudinal tumor assessment to help guide clinical decisions.

**Supplementary Information:**

The online version contains supplementary material available at 10.1186/s13073-024-01293-9.

## Background

Metastatic recurrence is the principal cause of death in breast cancer patients [1]. The classical “one step” Darwinian model of cancer evolution posits that metastases originate from rare primary tumor subclones that possess all of the properties needed to survive and traverse the metastatic cascade [2]. However, given the multifaceted selective pressures encountered by tumor cells during each step of the metastatic cascade (i.e., dissemination, intravasation, circulation, extravasation, localization, colonization, outgrowth, and treatment), it has been argued that rare genomic variation within primary tumors is unlikely to provide every type of fitness needed for disseminating tumor cells to give rise to an overt metastasis [3, 4]. Moreover, the observation that bulk gene expression patterns in primary tumors are associated with the probability of metastatic recurrence is difficult to reconcile with a model in which metastatic potential is attributable to rare cells within a primary tumor [3, 4]. These considerations helped spur alternate evolutionary models positing that metastatic potential is largely determined by the same pathway mutations that were responsible for primary tumorigenesis [4] and that further reinforcement of these pathways confers a metastatic advantage [3].

More recently, it has been proposed that metastatic recurrence is driven not by the generation and selection of new mutations, but rather by increased cellular plasticity propelled by latent transcriptional and epigenetic regulatory mechanisms [5]. Such models take flight from the relatively low frequency of recurrently mutated genes identified to date [5], with the exception of certain well-known mechanisms of treatment resistance such as mutations in *ESR1* [6], and hypothesize that new genomic alterations identified within metastatic recurrences only modestly affect tumor behavior.

In contrast to the above models, it remains possible that successful transversal of the metastatic cascade might instead require new genomic alterations that are acquired in a stepwise fashion *after* dissemination from the primary tumor. Indeed, we previously performed whole exome sequencing (WES) and shallow whole genome sequencing (sWGS) on primary tumors and metastatic tumors from the same patients to identify genomic determinants of breast cancer progression [7]. Compared with the primary tumors from which they arose, we found that treatment-refractory metastatic cancers preferentially harbored mutations and copy number alterations (CNAs) in several discrete genes and pathways [7]. These included four preferential CNAs (deletion of *STK11* and *CDKN2A*, amplification of *PAQR8* and *PTK6*), seven preferentially mutated genes (*ESR1*, *PALB2*, *MYLK*, *PEAK1*, *XIRP2*, *EVC2*, *SLC24ARG*), and 30 preferentially mutated pathways (e.g., mTOR, cell cycle, WNT, cAMP). While some of these alterations have been implicated in primary tumorigenesis (e.g., *STK11* loss, *CDKN2A* loss, *PALB2* mutation), others have not (e.g., *PAQR8* amplification and mutations in *MYLK*, *PEAK1*, *EVC2*, and *SLC2A4RG*). This pattern is exemplified by the selection for activating mutations in *ESR1*, which do not appear to play a role in the development of primary breast cancer, but rather in acquired resistance to anti-estrogen therapies [8].

The fact that each of the above genes and pathways was frequently and preferentially mutated in recurrent metastatic cancers at a significant level compared to their antecedent primary tumors implicates them as putative drivers of metastatic breast cancer progression [7]. Consistent with this, we demonstrated that preferentially mutated putative metastatic driver pathways, including mTOR, pRB, WNT, and PKA, exhibited concomitantly altered biochemical activities in metastases compared to their paired primary tumors of origin [7]. Furthermore, we recently demonstrated that PAQR8 is spontaneously upregulated and CN-gained in recurrent mouse mammary tumors that arise following therapy in a manner analogous to that observed in patients, promotes mammary tumor recurrence in mice, confers resistance to anti-estrogen therapy, anti-HER2 therapy, and chemotherapy, and is associated with poor overall survival as well as survival following recurrence in breast cancer patients [9]. These and other findings strongly suggest that genes and pathways that are frequently and preferentially altered in metastases compared to primary tumors may represent bona fide drivers of metastatic progression in breast cancer patients.

In light of evidence supporting their biological significance, our study raised the important question of whether mutations that preferentially occurred in metastatic recurrences, as determined by WES, truly arose after dissemination from the primary tumor or were instead already present within their respective primary tumors, but at frequencies too low to have been detected by WES. The answer to this question has important clinical implications since the ability to predict metastatic recurrence fundamentally depends on whether the accuracy of such predictions is limited solely by the sensitivity with which genomic heterogeneity can be assayed in primary tumors or, alternately, whether genomic alterations that contribute to metastatic recurrence may commonly be acquired *after* dissemination from the primary tumor. If true, this latter proposition would impose intrinsic limits on the ability to predict patient outcomes based solely on the properties of primary tumors.

Determining the stage of tumor progression at which the mutations contributing to metastatic recurrence are acquired would shed light on the extent to which metastatic potential is pre-encoded within primary tumors or is, instead, acquired after dissemination. This, in turn, would inform the question of whether predicting metastatic tumor behavior fundamentally requires the longitudinal assessment of tumors as they evolve over time.

Although traditional sequencing technologies have been used to evaluate whether metastatic driver mutations originate within primary tumors, these technologies are poorly suited for this task due to their limited sensitivity [10, 11]. For example, WES cannot reliably detect mutations occurring within subclones that constitute <50% of tumor cells (cancer cell fraction [CCF] < 0.50) [12]. Furthermore, while ultra-deep sequencing (UDS) has higher detection power, UDS alone is unable to detect tumor subclones that harbor mutations with variant allele frequencies (VAFs) below the error-floor, since this is dictated by the induction and propagation of errors during PCR amplification and corresponds to ~20% of tumor cells (VAF < 0.10, CCF $\lesssim$ 0.20) [12]. Conversely, while droplet digital PCR (ddPCR) can accurately detect mutations within rare subclones (1–20% of cells), this technology cannot comprehensively assess the broad repertoire of mutations that contribute to metastatic recurrence since only a small number of predefined mutations can be interrogated at a time. Further, all three of these approaches can be confounded by artifacts resulting from the formalin-fixed paraffin-embedded (FFPE) preservation method [13], which can greatly inflate the number of false-positive mutations detected.

In the present study, we sought to balance both depth (ability to detect rare subclones) and breadth (ability to detect many mutations in one assay), while also mitigating sequencing and preservation-related artifacts that determine the error-floor of traditional sequencing approaches. To do so, we coupled UDS with unique molecular identifier technology (UMI) to mitigate errors propagated by PCR, and with uracil-DNA glycosylase (UDG) treatment of tumor-derived DNA to mitigate errors induced by FFPE [14].

Using this combined UDS-UMI/UDG approach in paired primary and recurrent metastatic human breast cancers, we evaluated the extent to which putative driver mutations identified by WES as private to metastatic recurrences were nonetheless present in primary tumors within populations of tumor cells too small to be detected by WES. Our findings in 21 patients revealed that most mutations in putative metastatic drivers remained undetected within rare primary tumor subclones, even in cases where the rare ancestral primary tumor subclone was successfully identified. While sampling limitations inherent in retrospective analyses of primary tumors in patients cannot be ruled out as a factor contributing to the failure to detect some rare subclonal mutations, our findings—when combined with inferences from a probabilistic modeling framework—are consistent with the conclusion that most putative metastatic driver mutations were acquired after dissemination from the primary tumor and were likely to be of biological consequence.

Our results provide further evidence that metastatic progression is a mutationally driven, multi-step evolutionary process that extends beyond the point of primary tumor dissemination. Additionally, our study implicates several putative metastatic driver mutations, and the pathways they dysregulate, as potential therapeutic targets and emphasizes the importance of longitudinal genomic analysis during tumor progression to inform clinical decisions.

## Methods

### Analysis summary

Primers were designed and generated for two different panels using Qiagen QIAseq DNA V3 Panel Analysis and spanned 204 regions of interest (ROIs), each of which ranged in size from 11 to 22 bps. DNA samples were treated with UDG prior to library preparation to reduce the impact of FFPE-related sequencing artifacts. Libraries were prepared manually per manufacturer instructions and sequenced on a NextSeq 500 using 150 bp paired-end reads. Raw reads were aligned to hg19 and variants were called using smCounter [15] as implemented in Qiagen’s GeneGlobe Data Analysis Center.

The maximum amount of primary tumor tissue was assayed as was possible and feasible for archival samples obtained in the clinical setting. A total of 27 tumor blocks were assayed, consisting of three tumor blocks for one primary tumor, two tumor blocks for each of four primary tumors, and one block each for 16 primary tumors. All primary tumor blocks were FFPE-preserved. Tumor blocks contained approximately 1.5 cm^2^ (IQR = 0.8–2.0, min = 0.24, max = 4.0) of tumor material on the leading cutting side. In most cases, 10 sections at 10-micron thickness were cut from each tumor block and were used for DNA extraction (the exceptions being 4 tumor blocks in which 25, 20, 11, and 5 sections were available). DNA was treated with UDG prior to library preparation to reduce the impact of FFPE-related sequencing artifacts and five primary tumor blocks were re-sequenced in replicate to determine the effectiveness of UDG treatment.

The relative abundances of rare subclonal mutations across trinucleotide contexts were used to estimate the effectiveness of UDG treatment in mitigating FFPE-related artifacts. The detection of potential false positive mutations resulting from FFPE and/or other sequencing-related artifacts was assessed based on trinucleotide context, enrichment in the COSMIC database of mutations, background rare subclonal mutation burden, tumor block age, sequencing coverage, and number of mutations assayed. Univariate, multivariate, and combined likelihood models were used to estimate the probability that detection was inflated by false positives.

Detection power was assessed based on the smallest mutation cancer cell fraction (CCF) that could be reliably detected with 95% confidence. CCFs were estimated using locus-specific variant allele frequencies, allele-specific copy number, tumor ploidy and cellularity. Clonal composition was estimated using CCFs of mutations detected within rare primary tumor subclones. Mutations in major and minor subclones were defined as those with CCFs ≥ 0.50 and < 0.50, respectively. Mutations in rare subclones were defined as those with VAFs < 0.10, which approximately corresponds to mutations with CCFs $\lesssim$ 0.20 for a tumor with 100% cellularity and $\lesssim$ 0.40 for a tumor with 50% cellularity. Mutations detected in >50% of tumor cells (CCF > 0.50) could be inferred via the pigeonhole principle to have been sequentially acquired within the same clonal lineage.

Extended methods and corresponding citations [15–23] are provided in Additional file 1.

## Results

### Study design

In a previous study, several genes (e.g., *ESR1*, *PALB2*, *PEAK1*, *XIRP2*, *MYLK*, *SLC2A4RG*, *EVC2*) and pathways (e.g., mTOR, CDK/RB, WNT, cAMP) were identified that, based on gene set permutation and comparison to primary tumors, exhibited significantly more mutations within treatment-refractory metastases than expected by chance [7]. Considering that many of these mutations were detected by WES in metastatic recurrences, but not in their paired primary tumors of origin, these observations suggested the occurrence of non-random selection for mutations in these genes and pathways during metastatic progression, thereby implicating them as putative drivers of metastasis and/or treatment resistance. Since overt metastatic recurrence typically occurs in the context of treatment resistance, we refer to these mutations as “putative drivers of metastatic recurrence” or “putative metastatic driver mutations.” Although not all mutations defined in this manner would be expected to represent bona fide drivers of metastasis and/or recurrence, as opposed to passenger mutations, the statistical significance and high frequency of these putative metastatic driver mutations suggest the occurrence of biologically relevant selection for a substantial proportion of these mutations following dissemination.

In the present study, we used error-controlled ultra-deep sequencing coupled with FFPE artifact mitigation (UDS-UMI/UDG) in 21 patients (27 primary tumor blocks and 13 metastatic tumor blocks) to evaluate whether putative metastatic driver mutations were acquired after dissemination, as inferred from WES, or were instead present within rare subclones of the primary tumor that were undetectable by WES.

Patients included in this study were diagnosed with primary breast cancers between 1996 and 2013 (interquartile range [IQR] = 2007–2010). Receptor subtypes of patients were HR+ (90%, *n* = 19), HR+/HER2− (81%, *n* = 17), HR+/HER2+ (10%, *n* = 2), HR−/HER2− (10%, *n* = 2). All primary tumors were treatment-naïve at time of resection. In the adjuvant setting, 90% of patients had been treated with chemotherapy, 86% with endocrine therapy, 71% with radiation therapy, 10% with anti-HER2 therapy, and 5% with anti-CDK4/6 therapy. Patients developed overt metastasis at a median of 4.8 years after primary tumor diagnosis (IQR = 3.4–7.2 years, max = 17 years), with one patient having synchronous metastasis at 15 days. Thus, most putative metastatic driver mutations analyzed in this study occurred in the context of treatment-refractory, metastatic recurrences from HR+ primary tumors.

Two custom panels (Qiagen QIAseq DNA V3) were sequenced using UDS-UMI/UDG and covered 204 regions of interest (ROI) ranging in length from 11 to 22 bp (2395 total bases covered) within 145 genes (Additional file 2: UDS panel design). ROI included sites that are commonly mutated in both primary and metastatic tumors (e.g., *PIK3CA* and *TP53*) as well as sites of putative metastatic driver mutations that we previously identified as specific to the metastatic tumor by WES (e.g., *ESR1*) [7]. Sequencing of these panels enabled the assessment of 132 putative metastatic driver mutations at high read coverage (100 < MTs < 6K) within rare populations of cells in 21 antecedent treatment-naïve primary tumors. A median of 3 high-coverage putative metastatic driver mutations were assayed per primary tumor (IQR = 1–8, max = 33) (Additional file 2: MetSpec WES mutations), which was proportional (*R*^2^ = 0.76) to the total number of putative metastatic driver mutations that were detected in metastases, but not primary tumors, by WES (median = 9, IQR = 5–18, max = 66) (Additional file 1: Fig. S1).

The target amount of DNA (120 ng) assayed per tumor block per panel was reached for 84% of cases (median = 120, IQR= 120–181, min = 34, max = 240). Consequently, in most cases the theoretical maximum number of DNA molecules pre-amplification that could be assayed using our UDS-UMI/UDG approach was ~35,000. Panels were sequenced to a median depth of ~32,000 reads per site. A median of ~1300 unique molecular tags (MTs), which represent distinct pre-amplification DNA molecules, were deconstructed for each site from sequenced reads (see Additional file 1: Supplemental Methods). The median MT coverage at sites of putative metastatic driver mutations relevant to each primary tumor was 1160x (IQR = 599–2639x).

In parallel, UDS-UMI/UDG sequencing of metastatic tumors using the same panels enabled the reassessment of 73 mutations at high coverage within metastases that were previously called by WES. Thirteen tumor blocks representing core biopsies from 12 metastases were sequenced at a median depth of ~1000 reads per site, which resulted in a median of ~400 MTs per site. Two of 13 metastatic tumor blocks were FFPE-preserved.

Of the 28 patients from whom paired primary and metastatic tumors were assayed in our original study, primary tumors from 4 patients and metastatic tumors from 16 patients could not be reassessed due to depletion of tissue and DNA material. In addition, primary tumors from three patients assayed by UDS-UMI/UDG were excluded from analysis and not included in the above study characteristics due to lack of adequate coverage (<100 MTs) at putative metastatic driver mutation sites.

### Variant concordance and FFPE artifact mitigation

**Fig. 1:**
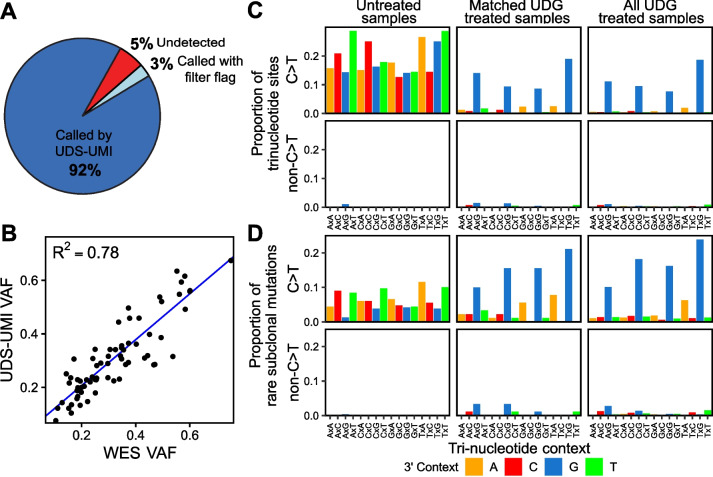
UDS-UMI/UMG variant calling is concordant with WES and mitigates FFPE-related sequencing artifacts. **A** UDS-UMI/UDG detected almost all mutations that were previously called in primary and metastatic tumors by WES. **B** Mutation VAFs were highly correlated between UDS-UMI/UDG and WES. **C, D** UDG treatment was effective in mitigating the majority of FFPE-related C>T artifacts from mutations called in rare subclones, as seen by the predominance of C>T rare subclonal mutations in untreated samples (*n*=5) compared with matched treated samples (*n* = 5) and with all treated samples (*n* = 30). **C** Considering trinucleotide context, a larger proportion of C>T covered sites exhibited rare subclonal mutations within untreated samples. **D** A larger proportion of rare subclonal mutations that were called within untreated samples occurred at C>T trinucleotide contexts. The 3’ (+1 position) nucleotide context of each mutation type is indicated by color

Seventy-three mutations previously called in metastases by WES were adequately covered by UDS-UMI/UDG in the same assayed metastatic tumor block. 95% (69/73) of these mutations were also called by UDS-UMI (Fig. 1A). VAFs of mutations that were called by both WES and UDS-UMI/UDG were highly correlated between the two technologies (*R*^2^ = 0.78, Fig. 1B), with VAFs determined from WES being slightly inflated compared to those from UDS-UMI.

Strikingly, treatment of tumor DNA with uracil-DNA glycosylase (UDG), which is used to digest uracil laden DNA that can result from FFPE-mediated cytosine deamination, was estimated to deplete rare subclonal variants resulting from FFPE artifacts by at least 88% (Fig. 1C, D) (Additional file 2: All UDS-UMI/UDG variants). Additional evidence supporting the bona fide nature of the rare subclonal mutations detected following UDG treatment is provided below and in the Supplemental Results (see Additional file 1).

### Variant detection power of UDS-UMI/UDG

**Fig. 2:**
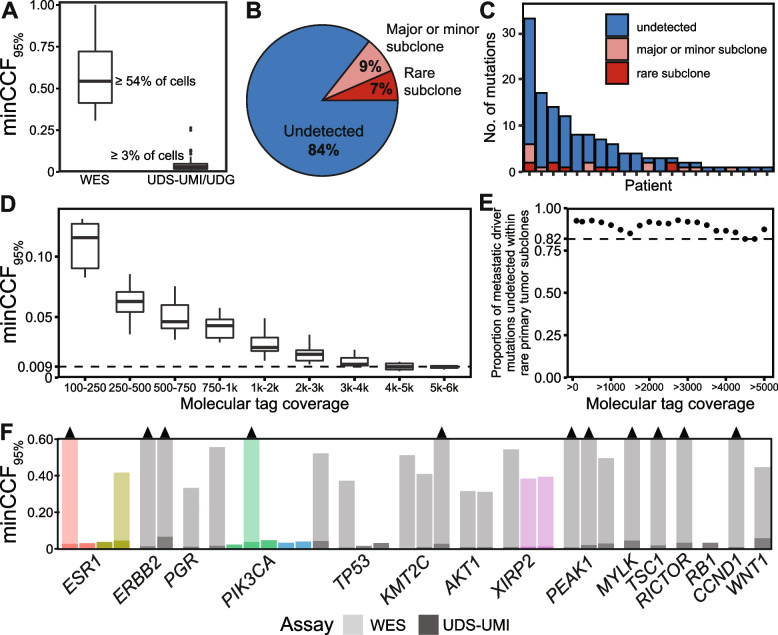
Most putative metastatic driver mutations were not detected within rare primary tumor subclones. **A** Detection power of WES and UDS-UMI sequencing technologies in terms of the minimum percent of cells in which the mutation must be present to be detected with 95% confidence (minCCF_95%_). **B** Proportion of metastatic driver mutations that were undetected within primary tumors by UDS-UMI/UDG versus those that were detected in major (≥50% of cells), minor (<50% of cells), or rare ($\lesssim$ 20% of cells) subclones. **C** The number of detected and undetected metastatic driver mutations within antecedent primary tumors in each patient assayed. **D** Detection power increased with higher molecular tag coverage, reaching a maximum of 0.9% of cells at >4k MT coverage. **E** Proportions of metastatic driver mutations that were undetected by UDS-UMI/UDG within rare primary tumor subclones as a function of molecular tag coverage. Limiting mutation sites to those with progressively higher coverage identifies a lower-bound of 82% of mutations that remain undetected, even at highest molecular tag coverage. **F** Detection power in primary tumors for UDS-UMI/UDG (dark bars) and WES (light bars) for a representative set of sites for mutations that were not detected by either technology. Mutations that were assayed in multiple regions of the same primary tumor, but were not identified, are labeled in colors other than grey. Black arrow heads indicate WES detection power > 60% of cells

As applied here, UDS-UMI/UMG had 95% power to detect mutations occurring in as few as 3.1% (median) of primary tumor cells (“minCCF_95%_” min = 0.0026, IQR = 0.016–0.049). In contrast, WES at 50× coverage was able to reliably call mutations at these same sites only if they occurred in greater than 50% of tumor cells (minCCF_95%_ median = 0.54, IQR = 0.41–0.72). Thus, UDS-UMI/UDG resulted in a 17-fold improvement in VAF/CCF sensitivity compared to WES (Fig. 2A) (Additional file 2: UDS MetSpec Detection).

Notably, of the 132 mutations identified by WES as private to metastases, 12 (9.1%) were detected by UDS-UMI in their corresponding primary tumor at relatively high VAF (≥ 0.10) and CCF (CCF median = 0.96, IQR = 0.77–1.2) (Fig. 2B, C). While the presence of each of these 12 mutations in primary tumors was suggested by WES, none met requisite filtering thresholds for variant calling despite their high VAF and CCF (i.e., each mutation had low coverage, a low number of variant reads, or an insufficient number of callers in consensus). The failure of WES to reliably detect these 12 high VAF mutations in primary tumors underscores its limited power to identify mutations, even those occurring within major subclones.

### Most putative metastatic driver mutations remain undetected in primary tumors by UDS-UMI

Despite the 17-fold increase in sensitivity afforded by UDS-UMI/UDG, 111 (93%) of the remaining 120 assayed putative metastatic driver mutations remained undetected within the primary tumor by UDS-UMI/UDG (Additional file 2: UDS MetSpec Detection) (Fig. 2B). In aggregate, 20 of 21 patients had at least one putative metastatic driver mutation that remained undetected in their primary tumor (Fig. 2C).

Importantly, sequencing coverage was not significantly different between sites of mutations that were detected versus those that were not (Additional file 1: Fig. S2A), suggesting that in most cases sequencing coverage likely maximized the variant detection sensitivity possible with UDS-UMI/UDG technology. Consistent with this, increasing sequencing coverage in order to increase detection power (Fig. 2D) failed to substantially increase the proportion of putative metastatic driver mutations that were detected in primary tumors (Fig. 2E). Indeed, even when assayed mutation sites were limited to those with the maximal detection power afforded by this technology (median minCCF_95%_ of 0.9% cells at sites with >4000 MTs) (Fig. 2D), 82% of putative metastatic driver mutations remained undetected (Fig. 2E). Thus, the failure to identify more than 80% of assayed putative metastatic driver mutations within rare subclones in their corresponding primary tumors could not be attributed to insufficient sequencing coverage or sub-optimal detection power.

Of the 120 putative metastatic driver mutations evaluated that were not detected in major subclones of the primary tumor by WES or UDS-UMI, 24 resided within a group of 117 genes widely implicated as likely drivers of breast cancer based on their significantly high frequencies of mutations and/or copy-number alterations within primary and/or metastatic breast cancer [6, 7, 24]. Strikingly, all but one (96%) of these mutations in likely breast cancer drivers remained undetected in rare subclones of primary tumors assayed by UDS-UMI. Undetected metastasis-specific mutations in likely breast cancer drivers included genes that are frequently mutated in primary breast cancer (*PIK3CA*, *TP53*, *KMT2C*, *AKT1, CCND1, CDKN1B, RB1, RHOA, RUNX1*) [24], as well as genes that have been reported to be frequently and preferentially mutated in metastases (*ESR1*, *ERRB2*, *XIRP2*, *PEAK1*, *MYLK, FLT4, RHOA, RICTOR, TP53*) [6, 7]. Each of these mutations occurred in genes with demonstrated pathogenic roles in cancer and included activating mutations in *ESR1* (D538G), *ERBB2* (V777L), *AKT1* (D323H), and *PIK3CA* (E542K), as well as inactivating mutations in *RB1* (N258 frameshift), *TP53* (V173L), and *CDKN1B* (S2 nonsense) [8, 25–28].

Beyond these 23 mutations in likely cancer drivers, and reflecting the high degree of mutational heterogeneity observed in human breast cancers, 96 putative metastatic driver mutations that were not detected in primary tumors by UDS-UMI occurred in genes encoding components of signaling pathways with demonstrated roles in cancer (e.g., *WNT1, FZD3, TSC1, JAK1, JAK2, JAK3, CCNE2, FBN1, INSR, LAMA1, NOTCH3, PIK3R2, PIK3CB, RELN*) [7]. In contrast to likely cancer drivers, which were previously implicated in breast cancer via mutation frequencies at the gene level [6, 7, 24], putative drivers of recurrent metastatic breast cancer were implicated at the pathway level based on their frequent, significant, and preferential mutation in recurrent metastatic tumors compared to primary tumors [7]. minCCF_95%_ for a representative set of putative metastatic driver mutations is visualized in Fig. 2F.

In summary, whether considering likely cancer drivers or putative metastatic drivers, the great majority of driver mutations identified in metastases remained undetected within rare subclones of the primary tumor by UDS-UMI. These findings are consistent with a model in which tumor cells in nearly all patients continue to undergo clinically relevant genomic evolution after their dissemination from the primary tumor via the acquisition of likely or putative metastatic driver mutations.

### Detection of putative metastatic driver mutations within rare primary tumor subclones

Nine putative metastatic driver mutations that were originally deemed by WES to be private to metastases were detected by UDS-UMI/UDG within rare subclones (i.e., VAF < 0.10) of their antecedent primary tumor (*JAK1* G902V, *LRP5* A65V, *PEAK1* K140Q, *CD14* E209K, *COL4A2* R1410Q, *SETD1B* E945K, *TGFBRAP1* S626F, *GHSR* V281I, and *NPRL3* S491L). In three patients (14%), two putative metastatic driver mutations were detected in their primary tumor. Thus, in six of 21 (29%) patients, at least one putative metastatic driver mutation was detected within a rare subclone of their primary tumor.

The CCF for each of the nine putative metastatic driver mutations that were identified within rare primary tumor subclones by UDS-UMI/UDG was estimated as a function of VAF, allele-specific copy number, tumor ploidy, and tumor cellularity (see Additional file 1: Supplemental Methods). Given that the number of alleles that were mutated (a.k.a. multiplicity factor, *s*) is unknown, CCFs were estimated for each possible *s* (Additional file 2: CCFs of visualized mutations).

**Fig. 3:**
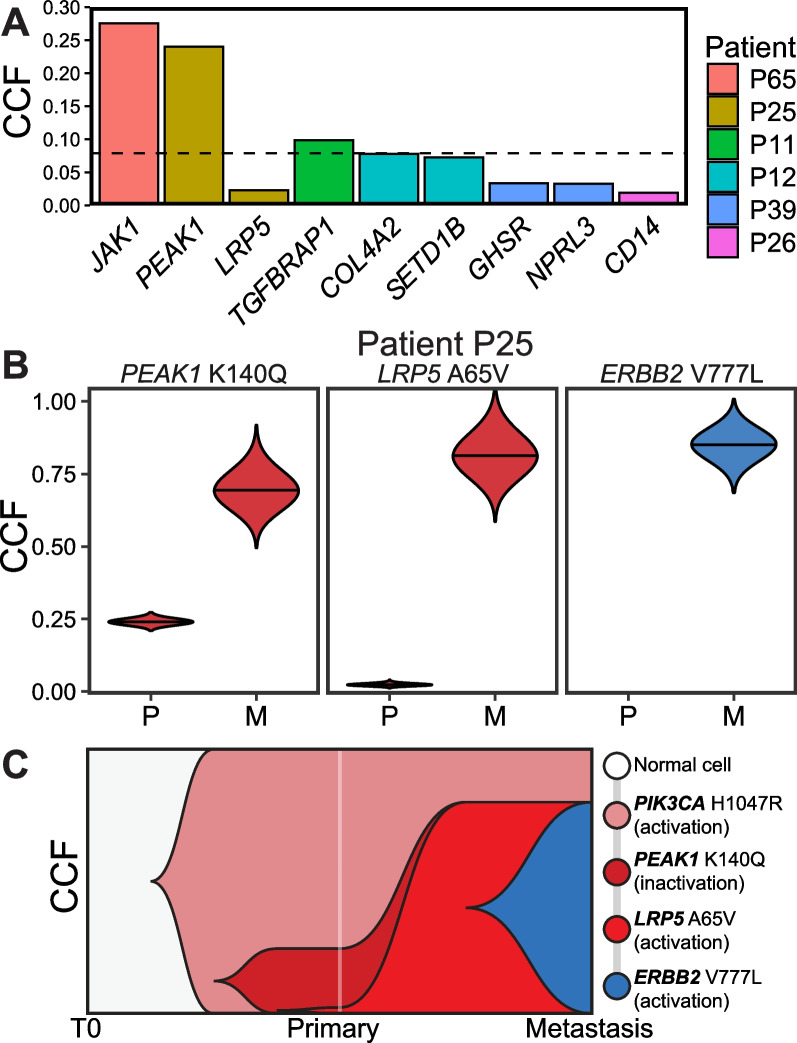
Putative metastatic driver mutations detected within antecedent rare primary tumor subclones inform modes of tumor evolution. **A** Estimated CCFs for metastatic driver mutations detected within rare primary tumor subclones using median CCF across possible multiplicity factors. Each color indicates a different patient. Horizontal dashed line indicates median CCF. **B** Within primary (“P”) and metastatic (“M”) tumors from patient P25, estimated posterior distributions of CCF (violin plots) for metastatic driver mutations that were either detected (*LRP5, PEAK1*) or undetected (*ERBB2*) by UDS-UMI/UDG within rare subclones of the primary tumor. Distribution height indicates the range of possible CCF values, with the widest point indicating the most likely estimate. For each mutation, there was only one allele that could be mutated. No violin plot distribution is shown for the *ERBB2* mutation in the primary tumor because the mutation was not detected in the primary tumor. **C** Muller plot showing the estimated evolutionary trajectories of metastatic driver mutations in patient P25

Putative metastatic driver mutations that were detected within rare subclones of primary tumors by UDS-UMI/UDG were estimated to have been present in a median of 7%, and as few as 2%, of primary tumor cells (CCF min = 0.019, median = 0.073, IQR = 0.033–0.099, max = 0.276, using median CCF across *s*; VAF min = 0.005, median = 0.021, IQR = 0.008–0.036, max = 0.087) (Fig. 3A). Two of these mutations exhibited modest CCFs despite having VAF < 0.10: *JAK1* G902V (UDS-UMI/UDG VAF = 0.087; CCF = 0.18, when *s* = 2; CCF = 0.37, when *s* = 1; median CCF = 0.28) and *PEAK1 K140Q* (UDS-UMI/UDG VAF = 0.062; CCF = 0.24, when *s* = 1). Both *JAK1* G902V and *PEAK1 K140Q* were originally detected within their respective primary tumors by WES, but at VAFs (0.063 and 0.024, respectively) below the threshold required for confident variant calling, again illustrating the inability of WES to reliably detect mutations in minor subclones.

### Evidence for the bona fide nature of putative metastatic driver mutations detected within ancestral rare primary tumor subclones

Results from multiple analyses indicated that the putative metastatic driver mutations detected within rare primary tumor subclones represent bona fide mutations rather than technical artifacts. In addition to the finding that sequencing coverage did not significantly differ between detected and undetected mutations (Additional file 1: Fig. S2A), multivariate and univariate regression analyses demonstrated that the ability to detect metastatic driver mutations within rare primary tumor subclones could not be attributed to background rare subclonal mutation burden (Kendall tau test* P* = 0.30, multivariate linear regression *P'* = 0.96), nor primary tumor block age (*P* = 0.85, *P'* = 0.59) (Additional file 1: Fig. S2B and S2C). These results suggest that mutation detection was unlikely to have been hampered by insufficient sequencing coverage or unmitigated FFPE artifacts, of which tumor block age is a known covariate [29].

Our analysis did reveal a greater probability of detecting a metastatic driver mutation within a primary tumor when more metastatic driver mutations were assayed (*P* = 0.0082, *P’* = 0.0051, Additional file 1: Fig. S2D). This result is consistent with at least two possibilities: 1) across patients a small percentage of metastatic driver mutations are present within rare primary tumor subclones; consequently, the probability of detecting these mutations would be proportional to the number of mutations assayed, or 2) metastatic driver mutations are not, in actuality, present within rare primary tumor subclones, but instead represent false-positives resulting from sequencing artifacts; consequently, the number of false-positive mutations detected would be anticipated to increase as more mutations are assayed.

To distinguish between these two possibilities, we employed a probabilistic model parameterized on the background burden of rare subclonal mutations (both panel-wide and trinucleotide-specific) and the number of assayed putative metastatic driver mutations. These were used to estimate the probability that the number of putative metastatic driver mutations detected within rare primary tumor subclones was the result of sampling artifactual mutations that randomly arose during sequencing. This analysis (Additional file 1: Table S1, Supplemental Methods) revealed that the number of putative metastatic driver mutations detected in rare subclones within their corresponding primary tumor of origin was greater than what would be expected based on either variation in background mutation burdens (combined likelihood 0.0012 ≤ *P* ≤ 0.023) or the number of putative metastatic driver mutations that were detected within unrelated primary tumors (sample permutation 0.022 ≤ *P* ≤ 0.029).

Together, these analyses provide evidence that putative metastatic driver mutations that were detected within rare primary tumor subclones by UDS-UMI/UDG represent bona fide mutations rather than randomly occurring sequencing artifacts. Consequently, these results indicate that UDS-UMI/UDG was able to identify mutations that arose within rare primary tumor subclones that had given rise to metastatic recurrences.

### Evaluation of ancestral rare primary tumor subclones identified by UDS-UMI/UDG suggests that putative drivers of metastatic recurrence are acquired both before and after dissemination

The identification of ancestral rare primary tumor subclones by UDS-UMI/UDG within roughly a third of patients permitted inferences to be made regarding the evolutionary trajectory of metastatic progression, as illustrated by patient P25 (Fig. 3B, C). For this patient, two putative metastatic driver mutations were detected within rare populations of cells within the primary tumor: a mutation in *PEAK1* (K140Q), which is a focal adhesion kinase involved in cell migration that is significantly and preferentially mutated in metastases [7], and a mutation in the WNT co-receptor, *LRP5* A65V.

Notably, *LRP5* A65V is structurally equivalent to a known activating mutation, *LRP5* G171V, that underlies autosomal dominant high bone density in two patient kindreds [30, 31], with A65V and G171V affecting equivalent residues on different blades of the first beta-propeller domain of LRP5 [32]. Based on this similarity *LRP5* A65V was first predicted, and then demonstrated, to abrogate the ability of Dkk1 to inhibit LRP5, thus resulting in increased WNT signaling activity [32].

Whereas *PEAK1* K140Q was present within a subclone representing 24% of tumor cells (CCF = 0.24, *s* = 1), *LRP5* A65V was estimated to be present in only 2% of cells in the primary tumor (CCF = 0.02, *s* = 1). Since the sum of their respective CCF distributions in the metastasis (CCF = 0.65 and 0.81, respectively) is significantly greater than 1 (*P =* 1.7 × 10^−6^), and since there was no ambiguity in multiplicity factors for either mutation (i.e., for each it was estimated that only one allele could possibly be mutated based on allele-specific copy number analysis), it can be deduced via the pigeonhole principle that this patient’s metastasis was seeded by a subclone bearing both mutations.

*PEAK1* is preferentially mutated in metastases, as are components of the focal adhesion pathway within which PEAK1 resides [7]. Similarly, the *LRP5* A65V mutation has been demonstrated to activate the WNT signaling pathway [32], which is also preferentially mutated in metastases [7]. Accordingly, evidence that the metastasis in patient P25 was seeded by a rare ancestral primary tumor subclone bearing putative metastatic driver mutations in both *PEAK1* and *LRP5* suggests that dysregulation of LRP5 and PEAK1 may have provided a metastatic advantage to mutant cells. This, in turn, further implicates these mutations as putative drivers of metastatic recurrence, rather than passenger mutations.

Intriguingly, the HR+/HER2− metastasis in this patient also harbored a known activating mutation in *ERBB2*, V777L [25], with concomitant loss-of-heterozygosity of *ERBB2* (Fig. 3B, C). This putative metastatic driver mutation was estimated to have arisen within this same *PEAK1/LRP5*-mutant subclone, yet it was not detected in the primary tumor by either WES or UDS-UMI/UDG (minCCF_95%_ = 0.013). These observations are consistent with the possibility that the *ERRB2* V777L activating mutation was acquired after dissemination of the rare *LRP5/PEAK1*-mutant primary tumor subclone that seeded the metastasis in this patient. Indeed, considering that this patient was treated with anti-estrogen therapy in the adjuvant setting, and that this same *ERBB2* activating mutation has been reported to arise in patients treated with anti-estrogen therapy [33], our results support a model in which the *ERBB2* V777L mutation was acquired and selected for after primary tumor dissemination in response to endocrine therapy administered following primary tumor resection.

Taken together, this constellation of findings suggests that in this patient 1) selection occurred via dissemination of a rare primary tumor subclone (2% of tumor cells) that had sequentially acquired two putative metastatic driver mutations (*LRP5* A65V and *PEAK1*), and 2) cells from this ancestral subclone acquired an *ERBB2* activating mutation after their dissemination from the primary tumor, ostensibly as a mechanism of resistance to treatment. Thus, the evolutionary trajectory of the metastasis in patient P25 is consistent with a multi-step, mutation-driven model of cancer progression wherein metastasis and treatment resistance result from iterative rounds of mutation acquisition and selection both before and after dissemination from the primary tumor.

**Fig. 4:**
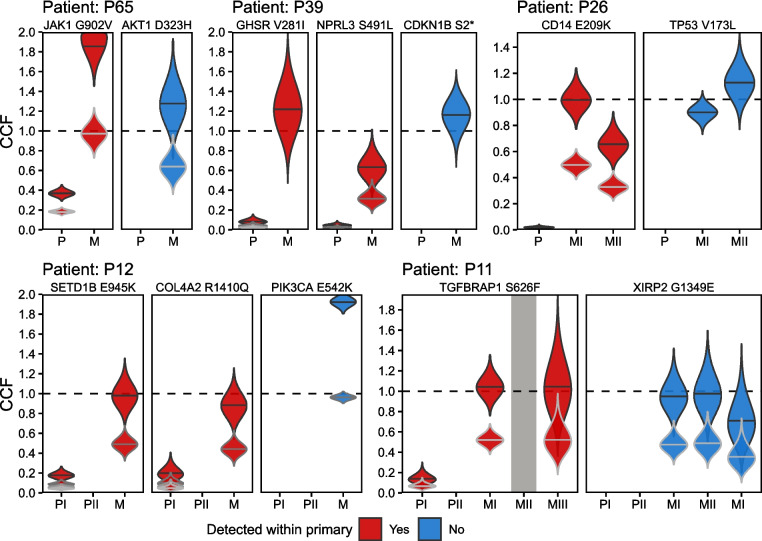
CCF estimates of putative metastatic driver mutations within primary and metastatic tumors. Estimated posterior distributions of CCF (violin plots) in primary tumor blocks (PI and PII) and metastatic tumor blocks (MI, MII, MIII) for (red) metastatic driver mutations that were detected by UDS-UMI/UDG within rare subclones of the primary tumor and (blue) metastatic driver mutations that were not detected within the primary tumor. Distribution height indicates the range of possible CCF values, with the widest point indicating the most likely estimate, using each possible number of mutant alleles (multiplicity factor). Dashed lines indicate a clonal mutation (CCF = 1). Within one metastatic tumor block in patient P11, one mutation did not reach required sequencing thresholds and is labeled in grey. No distributions are shown for mutations that were not detected within primary tumor blocks

Remarkably, in each of the 3 patients for whom two putative metastatic driver mutations were detected within a rare population of cells in their primary tumor, both newly detected putative metastatic driver mutations were estimated to have arisen within the same ancestral rare primary tumor subclone (Figs. 3B and 4). These included *LRP5* (WNT receptor) and *PEAK1* (focal adhesion kinase) within 2% of primary tumor cells in patient P25 (described above); *GHSR* (ghrelin growth hormone receptor) and *NPRL3* (mTORC1-regulating GATOR1 subunit) within 1.5–3% (depending on possible multiplicity factors) of primary tumors cells in patient P39; and *COL4A2* (a collagen involved in focal adhesion, relaxin signaling, and protein digestion and absorption pathways) and *SETD1B* (histone lysine methyltransferase) in 4–19% of primary tumor cells in patient P12. Furthermore, in patient P12 both putative metastatic driver mutations were detected in only one of two primary tumor blocks assayed. Thus, the implied spatial localization of detected putative metastatic driver mutations within a specific region of the primary tumor provides further evidence that multiple metastatic driver mutations can be acquired in the same rare subclonal lineage prior to dissemination.

In the three remaining patients, one putative metastatic driver mutation was detected within an ancestral subclone in each primary tumor, suggesting that it may have conferred an advantage for metastasis and/or treatment resistance. These included *JAK1* (JAK-STAT signaling) in 18–37% of primary tumor cells in patient P65, *TGFBRAP1* (TGF-beta receptor signaling) in 15–29% of primary tumor cells in P11, and *CD14* (surface antigen in the toll-like receptor signaling pathway) in 3% of primary tumors cells in patient P26.

The detection of nine putative metastatic driver mutations within rare ancestral primary tumor subclones in six patients implies that at least some mutations that confer a selective advantage for metastasis and/or treatment resistance do indeed exist within primary tumors prior to dissemination. Thus, beyond our initial evidence identifying putative metastatic driver mutations based on their frequent and preferential mutation in metastases compared to primary tumors [7], the apparent selection via dissemination of rare primary tumor subclones harboring these putative metastatic driver mutations further implicates these genes—and the pathways in which they reside—as biological contributors to metastatic recurrence.

Strikingly, for every patient in whom the ancestral rare primary tumor subclone was identified via UDS-UMI/UDG, evaluation of the associated metastasis revealed at least one additional putative metastatic driver mutation that was not detected in the primary tumor by either WES or UDS-UMI/UDG. To this end, within this same group of patients, 62 of 71 (87%) (median per patient = 86%, IQR = 84–90%) putative metastatic driver mutations deemed metastasis-specific by WES remained undetected even when that patient’s ancestral rare primary tumor subclone was identified by UDS-UMI/UDG. This observation provides further evidence that these additional putative metastatic driver mutations were acquired after dissemination.

Thus, the estimated evolutionary trajectory for tumors in each of the above six patients suggests that putative drivers of metastatic recurrence are most often acquired after dissemination from the primary tumor, which is consistent with a multi-step, mutation-driven model of cancer progression.

### Considerations on limitations of tumor sampling

Across patients, 93% of putative metastatic driver mutations and 96% of likely cancer driver mutations detected by WES in metastases, but not primary tumors, remained undetected by UDS-UMI/UDG within their antecedent primary tumor. This is consistent with a model in which tumor cells continue to acquire mutations in likely breast cancer drivers and putative drivers of metastatic recurrence after their dissemination from the primary tumor. However, while increasing the number of tissue blocks assayed per tumor failed to materially increase the number of putative metastatic driver mutations detected within rare primary tumor subclones (Additional file 1: Fig. S2E), it remains possible that putative metastatic driver mutations that failed to be detected by UDS-UMI/UDG resided within unassayed regions of the primary tumor, particularly considering the known topological heterogeneity of cancers [34, 35], and the relatively small amount of tumor tissue that is typically sequenced, or is available for sequencing, in studies of archival clinical material.

Nevertheless, considering the spatially localized heterogeneity known to exist in primary tumors, the finding that 87% of putative metastatic driver mutations remained undetected even when the ancestral rare primary tumor subclone that gave rise to the metastasis was identified provides additional evidence that the failure to detect these mutations was not wholly attributable to insufficient sampling of tumor material. Rather, their lack of detection by UDS-UMI/UDG within these ancestral rare primary tumor subclones is consistent with at least two additional possibilities: 1) that most if not all of these undetected putative metastatic driver mutations were acquired after dissemination from the primary tumor or, 2) that all of these mutations were present in the primary tumor within still smaller subclones nested within the identified ancestral rare primary tumor subclone. For the latter possibility to be true, and the former false, all undetected putative metastatic driver mutations in a given tumor would have to have been sequentially acquired (i.e., acquired in series within the same clonal lineage) within these hypothetical, exceedingly rare primary tumor subclones.

To evaluate the latter possibility, for each patient we estimated the upper bound of CCF in which this hypothetical ancestral primary subclone could have existed. For each patient, the estimated size of the detected ancestral primary tumor subclone determined the initial upper bound in the estimated size of a hypothetical subclone that had sequentially acquired each of the undetected putative metastatic driver mutations. Since in this scenario all undetected mutations would be present within the same subclonal lineage, upper bounds can be further decreased to the maximum detection power (minCCF_95%_) across undetected putative metastatic driver mutations.

For example, in patient P12 all 27 undetected putative metastatic driver mutations—including an inactivating 5-bp deletion frameshift mutation in *RB1* at N258—would have to have been sequentially acquired in, at most, 1% of primary tumor cells. Likewise, all 12 undetected putative metastatic driver mutations in patient P39—including an inactivating stop gain mutation in *CDKN1B* at S2—would have to have been sequentially acquired in, at most, 0.26% of cells. Similarly, patient P26 would have to have sequentially acquired all 11 undetected mutations in, at most, 0.66% of cells—including a known loss-of-function mutation in *TP53*, V147. Patient P65 would have to have sequentially acquired all 6 mutations in, at most, 0.91% of cells—including a known activating mutation in *AKT1* D323H. Patient P11 would have to have sequentially acquired all 5 mutations in, at most, 0.52% of cells, and patient P25 would have to have sequentially acquired the *ERBB2* activating mutation V777L in, at most, 1.3% of cells.

These considerations notwithstanding, while the ancestral subclone that gave rise to metastasis was identified in each of these cases, it still cannot be ruled out that all of the remaining undetected putative metastatic driver mutations were sequentially acquired within the same small identified ancestral primary tumor subclone, but were not contained within the tissue sample assayed. Nevertheless, the reported spatial localization of subclonal lineages [34, 35], coupled with the observation that 87% of putative metastatic driver mutations remained undetected in patients in whom the ancestral rare subclone was successfully identified, weigh against this possibility.

### A conceptual framework for the relative likelihoods of sequential mutation acquisition

The classical model of mutation acquisition and selection posits that a mutation is first acquired in one cell, which can then clonally expand due either to a selective advantage conferred by that mutation (i.e., a driver mutation), or to neutral evolution via random expansion of the clone independent of its relative fitness [35]. Clonal expansion to the point where a new mutation becomes represented in a substantial number of tumor cells thus provides an enlarged target population within which a subsequent mutation can occur. In this manner, mutations can be sequentially acquired and selected over time, resulting in multiple rounds of significant clonal expansion (i.e., >50% of tumor cells) and iterative changes to the mutational landscape shared by most tumor cells.

Based on the high clonal frequencies of putative metastatic driver mutations that we observed in metastases, but not their ancestral primary tumors, our findings to this point were consistent with a “driver model” in which multiple mutations that confer a metastatic advantage sequentially occur and are selected for after dissemination from the primary tumor. Nevertheless, we considered the null hypothesis that all mutations deemed specific to metastases by WES and UDS-UMI/UDG were passenger mutations (“passenger model”) that had no impact on tumor behavior, and that these mutations were already present within rare populations of primary tumor cells since this latter possibility cannot be ruled out due to the inability to assay all tumor cells in a clinical setting. For the passenger model to be true, all mutations deemed specific to metastases would have to have been sequentially acquired within the same subclonal lineage in the primary tumor.

To evaluate these alternative models, we developed a conceptual framework based on a classical model of mutation acquisition and selection to estimate the probability that a series of ($d$) driver mutations in a series of $\left(k\right)$ mutations was sequentially acquired within the same clonal lineage (${P}_{sequence}\left(k, d\right)$), as a function of mutation rate (μ), tumor size ($T$), and the extent to which clonal expansion occurred following each mutation ($c$). As derived in Additional File 1: Supplemental Results, this probability was estimated using the combined likelihood across $k$ Poisson processes, which can be expressed as:

$${P}_{sequence}\left(k, d\right)\propto {\left(1- {\left(1-\mu \right)}^{Tc}\right)}^{d}\times {\mu }^{k-d-1}\times (1- {\left(1-\mu \right)}^{T})$$

This relationship indicates that the probability of a tumor subclone acquiring a series of mutations exponentially decreases with greater numbers of mutations, smaller numbers of driver mutations, lesser extents of clonal expansion, lower mutation rates, and smaller tumor sizes. A core inference from this relationship is that the probability of acquiring a series of mutations within the same clonal lineage is substantially higher for driver mutations than for passenger mutations, because each driver mutation in the series is more likely to result in clonal expansion, which gives rise to a larger pool of cells within which a subsequent mutation can potentially be acquired (via a Poisson process wherein the number of trials is determined by the number of cells).

Conversely, the probability of acquiring a series of passenger mutations within the same clonal lineage is substantially lower than for driver mutations because the random nature of clonal expansion in this context entirely offsets any increase in the number of cells in a potentially expanded subclone. Mathematically, this probability became equivalent to the probability of at least one cell acquiring an initial mutation multiplied by the probability of acquiring the remaining $k-1$ passenger mutations in that same cell in the absence of clonal expansion (${\mu }^{k-1}$) (see Additional file 1: Supplemental Results). Consequently, the probability that a series of mutations acquired within the same clonal lineage consisted of passenger mutations, rather than driver mutations, becomes exponentially less likely as the number of mutations increases and the mutation rate decreases.

We next sought to quantify the relative likelihood of the passenger model versus the driver model based on observed data—namely, the number of mutations that were estimated by WES and UDS-UMI/UDG to be metastasis-specific and sequentially arising (CCF > 0.50), as well as the extent of clonal expansion anticipated for each of the models. We found that patients exhibited, on average, 60 sequentially arising metastasis-specific coding mutations as identified by WES, of which an average of 8 were putative metastatic driver mutations. Of these, an average of 3.4 of were assayed by UDS-UMI/UDG (i.e., a minority of putative metastatic driver mutations were assayed by this method) and deemed metastasis-specific due to their lack of detection within the primary tumor. These values for the identified number of sequentially arising metastasis-specific mutations ($k$ = 60, 8, and 3.4) were incorporated into the above probabilistic framework.

Incorporating these values for $k$ into the above framework indicated that the passenger model was far less likely to be true than the driver model (relative likelihoods of ~3.2 × 10^−266^, ~3.2 × 10^−32^, and ~1.6 × 10^−11^, respectively). Notably, this was the case even if we assumed for the driver model that only a third of identified mutations were actually drivers (relative likelihoods of 10^−90^, 10^−12^; ~7.9 × 10^−6^, respectively) (see Additional file 1: Supplemental Results). This suggests that the numbers of metastasis-specific mutations observed were far more likely to have arisen via non-random selection and significant clonal expansion due to at least a subset of these mutations being driver mutations. Given that these mutations were not detected within the primary tumor by UDS-UMI/UDG, these findings strongly imply that the sequential acquisition and clonal expansion of these putative driver mutations most likely occurred after their dissemination from the primary tumor.

We also evaluated an alternative hypothesis that metastasis-specific mutations were indeed driver mutations, but were sequentially acquired within the primary tumor in the context of non-random, yet insignificant, clonal expansion, which would be a required element of this model given that these mutations were detected neither by WES (detection power >50% of cells) nor UDS-UMI/UDG (detection power >3% of cells). For each patient, we used for $k$ the total number of coding mutations that were identified by WES as being metastasis-specific and sequentially arising, and for $d$ the subset of those that were putative metastatic driver mutations.

Strikingly, our estimates indicated for every patient that it was highly unlikely (i.e., <10^−11^) that the metastasis-specific mutations identified had, in actuality, been sequentially acquired in a subclonal lineage comprising no more than 50% of cells within the primary tumor, even when offset by the non-random clonal expansion conferred by driver mutations. Moreover, when considered across patients, this combined likelihood approached zero. Additionally, when $$k$$ and $$d$$ were specified as the number of sequentially arising, metastasis-specific putative metastatic driver mutations that remained undetected by UDS-UMI (i.e., $$k$$ = $$d)$$, we estimated for patients with 3 or more undetected sequentially acquired, metastasis-specific mutations that it was highly unlikely (range 0.29–2.2 × 10^−9^) that these mutations could have sequentially arisen within the primary tumor while remaining beneath the detection floor of UDS-UMI (average of 3% of cells)—even when offset by the non-random clonal expansion conferred by driver mutations. Moreover, when considered across all patients, the combined likelihood was 3.1 × 10^−28^, rendering this alternative hypothesis exceedingly unlikely. Importantly, the combined likelihood across the six patients for whom the ancestral rare primary tumor subclone that gave rise to the metastasis was identified was also exceedingly low (1.6 × 10^−23^), thereby providing further evidence that the low probabilities observed across patients was not simply the result of sampling limitations.

Lastly, due to the far lower relative likelihood of the passenger model compared with the driver model based on the above considerations, when examining primary tumors we would expect to find far fewer passenger mutations, compared to driver mutations, that were sequentially acquired within the same clonal lineage. Consequently, if the passenger model is correct (i.e., all metastasis-specific mutations were passenger mutations that were sequentially acquired within the primary tumor), we would expect to identify far fewer sequentially acquired metastasis-specific mutations compared to sequentially acquired truncal mutations (i.e., mutations acquired in the primary tumor in the context of significant, non-random clonal expansion due to the driver function of many of these mutations).

Strikingly, and contrary to this passenger model-based prediction, we found that patients exhibited, on average, 3 times more (mean = 3.1×, median = 1.2×, IQR = 0.4–4.9×, max = 10×) sequentially arising metastasis-specific mutations than truncal mutations, which is far greater than the ratio of metastasis-specific:truncal mutations predicted by the passenger model (i.e., <<1).

In aggregate, these and other findings (see Supplemental Results) provide evidence that the probability that all observed metastasis-specific mutations were, in actuality, passenger mutations that had been sequentially acquired in the primary tumor in the absence of non-random and significant clonal expansion is exceedingly low compared to a model in which driver mutations are acquired after dissemination that result in significant and non-random clonal expansion. This, in turn, argues against a passenger model in which all putative metastatic driver mutations that were newly detected in metastases either provided no selection advantage or were sequentially acquired within rare ancestral primary tumor subclones in the absence of clonal expansion. Similarly, our findings across patients also argue against the possibility that metastasis-specific mutations were driver mutations that were sequentially acquired within the primary tumor in the absence of significant clonal expansion, such that they remained beneath the detection floor of UDS-UMI.

## Discussion

According to Laplacian determinism, all future states of a complex system can theoretically be predicted if the current state of that system is measured at a sufficiently high resolution. While advances in genomic sequencing technologies have increased the resolution at which the initial state of breast cancer (i.e., the primary tumor) can be defined, the extent to which these advances have brought the prediction of future states, such as metastatic recurrence, closer to this Laplacian ideal is unclear.

The ability to predict metastatic recurrence, as it relates to the question of Laplacian determinism, hinges on whether predictive power is limited solely by the sensitivity with which genomic heterogeneity in primary tumors can be assayed or, instead, whether a significant fraction of the genomic alterations that drive metastatic recurrence are acquired after primary tumor dissemination, since this would place inherent limitations on the ability to predict patient outcomes based solely on analysis of the primary tumor. For these reasons, determining the stage of tumor progression at which the mutations driving metastatic recurrence are acquired should inform whether accurately predicting future tumor behavior in patients fundamentally requires longitudinal genomic assessment beyond the primary tumor.

In this study, we used UDS-UMI/UDG technology to detect the presence of putative metastatic driver mutations in their antecedent primary tumors at an extremely high level of sensitivity (95% confidence to detect mutations present in a median of ~3% of cells, maximal reliable detection at 1% of cells). Our findings suggest that 1) the great majority (93%, lower-bound 82%) of putative metastatic driver mutations occurred after primary tumor dissemination; 2) nearly a third of patients harbored rare primary tumor subclones that appear to have possessed enhanced metastatic potential (i.e., they successfully seeded metastatic tumors despite their low abundance in primary tumors); and 3) even in those instances in which the rare primary tumor subclone that had seeded a metastasis was identified, most putative metastatic driver mutations still appeared to have been acquired within the same subclone after dissemination from the primary tumor, including likely driver mutations in *ESR1*, *ERBB2*, *AKT1*, *PIK3CA*, *RB1*, *TP53*, and *LRP5*. Our findings collectively argue that breast cancer progression resulting in metastatic recurrence is a multi-step process that is dependent on the sequential acquisition of additional genomic alterations.

In addition, we present a conceptual framework coupled with individual patient genomic data indicating that it is highly unlikely that all of the putative metastatic driver mutations estimated to have been newly acquired within metastases in a sequential fashion were instead sequentially acquired within their corresponding primary tumors in the absence of non-random and significant clonal expansion, which would be required given the failure to detect these mutations in primary tumors using the highly sensitive UDS-UMI/UDG assay. Consequently, this framework suggested that it was highly unlikely that all metastasis-specific putative metastatic driver mutations were either passenger mutations, whether acquired before or after dissemination, or pre-existing driver or passenger mutations within primary tumors that became detectable solely due to the expansion of their subclones.

While insufficient sampling of the primary tumor, which is an inherent limitation of virtually all retrospective analyses of archival clinical tissue, cannot be ruled out as a factor contributing to the failure to detect putative metastatic driver mutations in primary tumors, the fact that estimated probabilities remained extremely low even when restricted to patients for whom the ancestral rare primary tumor subclone that gave rise to the metastasis was identified weighs against the possibility that the low probability observed across patients was simply the result of sampling limitations. Thus, these modeling considerations further support metastatic recurrence as a mutation-driven multi-step process that extends beyond the point of primary tumor dissemination.

To the extent that our findings suggest the existence of rare subclones within primary tumors that possess enhanced metastatic potential, their presence is reminiscent of the classical Darwinian model of cancer evolution in which metastases originate from rare primary tumor subclones that possess all of the properties needed to survive each step of the metastatic cascade. However, even in those patients harboring such rare primary tumor subclones, our evidence indicates that it was nearly universally the case that additional putative metastatic driver mutations were acquired after primary tumor dissemination. Thus, while it appears that enhanced metastatic potential is likely to be conferred by mutations present within the primary tumor, our findings argue against both the classical “one-step” Darwinian model of cancer evolution from a minor subclone, and also against models in which metastatic potential is predetermined in its entirety by the same driver mutations that were responsible for primary tumor formation. Instead, our results support a model in which the ability of tumor cells to survive and traverse the evolutionary bottlenecks and repeated rounds of selection encountered during the metastatic cascade requires the stepwise acquisition of new genomic alterations, which predominantly occurs after dissemination from the primary tumor.

It bears noting that the minority of cases in which putative metastatic driver mutations were detected within rare subclones in the antecedent primary tumor by UDS-UMI/UDG provide orthogonal evidence that these mutations were indeed drivers of metastatic recurrence, given their apparent selection. For example, an *LRP5* activating mutation and a *PEAK1* mutation originally found solely in a metastasis were subsequently detected within the primary tumor by UDS-UMI/UDG and estimated to be present within the same rare subclone comprised of only 2% of tumor cells. This suggests that the metastatic potential of this primary tumor may have been determined by combined dysregulation of the WNT and focal adhesion pathways within a rare subclone, rather than being a characteristic possessed by all, or even most, cells of the primary tumor.

In an analogous manner, mutations in *GHSR* and *NPRL3*—each of which function within the mTOR signaling pathway—were identified within the same rare primary tumor subclone (2% of cells) in a second patient. Consistent with this, we previously determined that mTOR pathway activity is elevated in metastatic tumors compared to primary tumors and that significant increases in mTOR activity were associated with two or more mutations in the mTOR signaling pathway, which was frequently seen in metastases but not primary tumors [7]. Accordingly, the identification of two mTOR pathway mutations within the same metastasis-seeding rare primary tumor subclone provides additional corroborating evidence that multiple mutations in the mTOR pathway may additively and/or synergistically promote metastatic recurrence. More generally, this observation suggests that the increased metastatic potential apparently conferred by these mutations was restricted to a rare population of cells within the primary tumor, rather than being a shared characteristic of most, much less all, primary tumor cells.

A principal conclusion of this study is that UDS alone is insufficient to accurately quantify the presence of mutations within rare primary tumor subclones. Rather, UMI technology is required to distinguish sequencing artifacts that occur after the PCR amplification of genuine DNA variants, and UDG treatment of FFPE-derived DNA is required prior to sequencing to mitigate artifacts that commonly result from this standard preservation method. Indeed, while deep sequencing performed following WES in a study of six paired breast cancer metastases [36] allowed for the more accurate estimation of mutation clonality and provided increased power to detect mutations within primary tumors compared to WES alone, this approach was unable to detect rare subclonal mutations occurring beneath the error-floor attributed to standard sequencing approaches (5–10% of reads). Thus, while some prior studies have identified metastatic tumor mutations within antecedent primary tumors [6, 36, 37], the technological approach employed here provides substantially greater sensitivity, thereby enabling additional biological insights into the role played by rare genomic heterogeneity within primary tumors in the course of metastasis and treatment resistance.

Notably, Razavi et al. also sought to determine whether mutations suggested by WES to be newly acquired in metastases pre-existed within rare subclones in primary tumors by using the MSK-IMPACT hybridization panel, which was capable of identifying mutations in ~350 canonical cancer-related genes in as few as 1.3% of cells [6]. They, too, found that clinically relevant mutations that were detected in metastases by targeting sequencing, such as mutations in *ERBB2*, *EGFR*, *NF1*, *KRAS*, and *MYC*, were often not detected within rare subclones of the original primary tumor using the MSK-IMPACT hybridization panel. While the MSK-IMPACT panel is a powerful tool for the identification of mutations that may be clinically actionable [38], this panel is focused on canonical cancer-related genes, rather than the larger repertoire of genes that are preferentially mutated in metastatic breast cancers. Since our prior findings indicated that the majority of genomic alterations that preferentially occurred in metastases had not previously been implicated in primary tumorigenesis [2], a strength of our study is that it sought to quantify the frequency with which ostensibly private metastatic driver mutations were, in actuality, present within rare subclones of their antecedent primary tumor—a question that cannot be addressed using a panel focused on canonical cancer-related genes. Furthermore, our study is unique in that UDG treatment was used prior to sequencing to mitigate FFPE-related artifacts that could otherwise inflate the frequency at which mutations are erroneously detected within rare primary tumor subclones. Notwithstanding these differences in approach, our findings are consistent with those reported by Razavi et al.

Despite the relative strengths of our study, there are limitations. First, while UDG treatment was estimated to have removed >88% of FFPE sequencing artifacts, complete elimination of these artifacts was almost certainly not achieved. Second, sampling error is an unavoidable limitation of studies of the genomic evolution of cancer in that it is rarely, if ever, possible to comprehensively assay all regions within primary tumors in a clinical setting. Accordingly, a putative metastatic driver mutation that was present in the antecedent primary tumor may nevertheless go undetected due to spatial heterogeneity within the tumor or the small size of the subclone. In either case, assaying a greater number of regions within the primary tumor would be anticipated to result in improved estimates for clonality, along with the models based upon them.

It must also be noted that the metastatic cohort analyzed consisted primarily of HR+ patients who had previously been treated in the adjuvant setting (i.e., after primary tumor resection). Accordingly, genomic analysis of paired primary tumors and metastases with and without exposure to therapy would be required to deconvolute the contribution of newly acquired mutations to treatment resistance versus metastasis per se. Similarly, future studies will be required to determine whether patterns of mutation acquisition revealed in this study are recapitulated within patients with the triple-negative breast cancers, a subtype that is characterized by increased genomic heterogeneity [39]. Nevertheless, since the vast majority of tumors analyzed were from early stage HR+ patients who received systemic treatment prior to metastatic recurrence, the samples analyzed closely reflect the clinical context for most breast cancer patients.

Lastly, the conceptual framework used to estimate the relative likelihood of the passenger versus driver models is, by design, oversimplified insofar as it omits several factors likely to influence the dynamics of mutation acquisition and clonal expansion, including rates of cell proliferation and cell death, changes in mutation rate and tumor size over time, and the sensitivity and specificity of detecting bona fide mutations by WES and UDS-UMI. Nevertheless, we anticipate that the relative probabilities of mutations being sequentially acquired in the context of random (i.e., passenger) versus non-random (i.e., driver) clonal expansion can still be meaningfully compared without estimating the magnitudes of these additional parameters, since these would ostensibly be shared by both models.

For example, while the use of different rates of mutation generation in our conceptual framework does affect the probability estimate for a given mutation acquisition model, it does not change the resulting conclusion that the passenger model is orders of magnitude less likely than the driver model. Indeed, even if a high exomic mutation rate is assumed (0.1 mutations per sequenced exome per cell division) [40], the probability of acquiring a series of only passenger mutations still quickly approaches zero with higher numbers of mutations (e.g., <0.001 for 4 mutations, <0.0001 for 5 mutations). Similarly, the higher mutation rate expected for passenger mutations compared with driver mutations is offset by the lack of non-random and significant clonal expansion associated with passenger mutations, since these aspects of clonal expansion greatly increase the probability of sequential mutation acquisition. Our conclusions are further supported by independent modeling studies indicating that the increase in tumor growth attributable to driver mutations renders their sequential acquisition increasingly more probable compared to passenger mutations over longer periods of time [41].

An additional limitation of the conceptual framework employed is that mutation generation events are considered independent, thereby allowing for a relatively simple formulation of the logic underlying each of the opposing models. However, the probability of acquiring a new mutation within a series of mutations fundamentally depends on the extents of clonal expansion for each preceding mutation in the series. For example, while order is not specified in the formulated model due to the postulated independence of mutation generation events, it is nevertheless apparent that an alternating sequence of passenger and driver mutations is far more likely to occur (e.g., PDPDPDPD) than a non-alternating sequence (e.g., PPPPDDDD) because of the increasingly low likelihood of acquiring several passenger mutations in sequence, yet our model treats each scenario the same. Thus, even if order was specified in the framework, doing so would only increase the relative likelihood of the driver model compared with the passenger model.

Finally, we considered a scenario in which more than one mutation is generated simultaneously such that mutations do not represent independent events. For example, mutagenesis via kateagis, wherein multiple mutations are generated simultaneously within a hyper-focal region (~100s of bps), could have inflated the number of independent mutational events assessed in our model [42]. However, we found that only ~3% of mutations identified by WES were localized, occurring within 1kbp of another mutation. This implies that simultaneous mutational events are relatively rare and, thus, would not materially affect estimates derived from this conceptual framework.

## Conclusions

In this study, error-controlled ultra-deep sequencing coupled with FFPE artifact mitigation with a maximum mutation detection sensitivity of ~1% of tumor cells was used to assess the presence of 132 WES-identified “metastasis-specific” mutations within antecedent primary tumors from 21 patients. We found that 93% of metastasis-specific mutations in putative metastatic driver genes remained undetected within primary tumors, as did 96% of metastasis-specific mutations in genes encoding known breast cancer drivers. Strikingly, even for the 29% of patients in whom the ancestral primary tumor subclone responsible for seeding the metastasis was identified, 87% of metastasis-specific putative driver mutations remained undetected.

These findings were supported by a conceptual framework incorporating genomic data for individual patients indicating that a “driver” model in which mutations that cause non-random and significant clonal expansion are acquired after dissemination is exceedingly more likely than a “passenger” model in which metastasis-specific putative metastatic driver mutations either provide no selection advantage or are sequentially acquired within rare ancestral primary tumor subclones in the absence of significant clonal expansion, such that they remain beneath the detection floor of UDS-UMI.

Together, findings from this study strongly suggest that putative metastatic driver mutations are sequentially acquired and selected within the same clonal lineage before, but to a greater extent after, dissemination from the primary tumor, and that these metastasis-specific mutations are bona fide drivers of metastatic recurrence. Thus, despite inherent limitations in sampling archival primary tumors, our results indicate that tumor cells in most patients continue to undergo clinically relevant genomic evolution after their dissemination from the primary tumor. In doing so, findings from this study argue against “one step” evolutionary models in which metastases originate from rare primary tumor subclones possessing all of the properties needed to traverse the metastatic cascade, and also against models in which metastatic potential is entirely pre-encoded within primary tumors by the same pathway mutations that were responsible for primary tumorigenesis.

Finally, in providing evidence for the common acquisition and selection of putative metastatic driver mutations after dissemination, our findings offer further support that metastatic recurrence in breast cancer is a multi-step, mutation-driven process. This ongoing genomic evolution highlights the impossibility of predicting metastatic behavior based solely on the analysis of primary tumors, which in turn emphasizes the importance of longitudinal tumor assessment to identify newly arising mutations that may drive metastatic recurrence in order to help guide clinical decisions.

### Supplementary Information

**Additional file 1.** Contains the sections Supplemental Methods, Supplemental Results, and Supplemental Figures.

**Additional file 2.** Contains data on the regions-of-interest assayed in each UDS-UMI/UDG panel (“UDS panel designs”), all variants called by UDS-UMI/UDG (“All UDS-UMI/UDG variants” and “UDS-UMI/UDG data dictionary”), metastasis-specific mutations as determined by WES (“MetSpec WES mutations”), information on the detection (or lack thereof) of metastasis-specific putative metastatic driver mutations within primary tumors (“UDS MetSpec Detection”), and CCFs of putative metastatic driver mutations used in Figs. 3 and 4 (“CCFs of visualized mutations”).

## Data Availability

Panel ROIs, putative metastatic driver mutations that were originally deemed to be specific to metastases by WES, as well as all variants called by UDS-UMI/UDG are included in Additional file 2. Raw WES and sWGS data were first described in (7) and are publicly accessible in the NCBIBioProject, PRJNA610817 https://www.ncbi.nlm.nih.gov/bioproject/?term=PRJNA610817.

## References

[1] Torre LA, Siegel RL, Ward EM, Jemal A (2016). Global Cancer Incidence and Mortality Rates and Trends—An Update. Cancer Epidemiology Biomarkers Prevention..

[2] Fidler IJ, Kripke ML (1977). Metastasis results from preexisting variant cells within a malignant tumor. Science..

[3] Vanharanta S, Massagué J (2013). Origins of metastatic traits. Cancer Cell..

[4] Bernards R, Weinberg RA. Metastasis genes: A progression puzzle. Nature. 2002;418(6900):823. 10.1038/418823a 12192390

[5] Garcia-Recio S, Hinoue T, Wheeler GL, Kelly BJ, Garrido-Castro AC, Pascual T (2023). Multiomics in primary and metastatic breast tumors from the AURORA US network finds microenvironment and epigenetic drivers of metastasis. Nature Cancer..

[6] Razavi P, Chang MT, Xu G, Bandlamudi C, Ross DS, Vasan N (2018). The Genomic Landscape of Endocrine-Resistant Advanced Breast Cancers. Cancer Cell..

[7] Paul MR, Pan TC, Pant DK, Shih NNC, Chen Y, Harvey KL, et al. Genomic landscape of metastatic breast cancer identifies preferentially dysregulated pathways and targets. J Clin Invest. 2020;130(8):4252–65. 10.1172/JCI129941 PMC7410083 32657779

[8] Jeselsohn R, Buchwalter G, De Angelis C, Brown M, Schiff R (2015). ESR1 mutations—a mechanism for acquired endocrine resistance in breast cancer. Nature Reviews Clinical Oncology..

[9] Chen S, Paul MR, Sterner CJ, Belka GK, Wang D, Xu P (2023). PAQR8 promotes breast cancer recurrence and confers resistance to multiple therapies. Breast Cancer Research..

[10] Kroigard AB, Larsen MJ, Brasch-Andersen C, Laenkholm AV, Knoop AS, Jensen JD (2017). Genomic Analyses of Breast Cancer Progression Reveal Distinct Routes of Metastasis Emergence. Sci Rep..

[11] Takeshita T, Yamamoto Y, Yamamoto-Ibusuki M, Inao T, Sueta A, Fujiwara S (2015). Droplet digital polymerase chain reaction assay for screening of ESR1 mutations in 325 breast cancer specimens. Translational Research..

[12] Shi W, Ng CKY, Lim RS, Jiang T, Kumar S, Li X (2018). Reliability of Whole-Exome Sequencing for Assessing Intratumor Genetic Heterogeneity. Cell Reports..

[13] Kim H, Lee AJ, Lee J, Chun H, Ju YS, Hong D (2019). FIREVAT: finding reliable variants without artifacts in human cancer samples using etiologically relevant mutational signatures. Genome Medicine..

[14] Berra CM, Torrezan GT, de Paula CA, Hsieh R, Lourenço SV, Carraro DM (2019). Use of uracil-DNA glycosylase enzyme to reduce DNA-related artifacts from formalin-fixed and paraffin-embedded tissues in diagnostic routine. Applied Cancer Research..

[15] Xu C, Nezami Ranjbar MR, Wu Z, DiCarlo J, Wang Y (2017). Detecting very low allele fraction variants using targeted DNA sequencing and a novel molecular barcode-aware variant caller. BMC Genomics..

[16] Alexandrov LB, Nik-Zainal S, Wedge DC, Aparicio SAJR, Behjati S, Biankin AV (2013). Signatures of mutational processes in human cancer. Nature..

[17] Letouzé E, Shinde J, Renault V, Couchy G, Blanc JF, Tubacher E (2017). Mutational signatures reveal the dynamic interplay of risk factors and cellular processes during liver tumorigenesis. Nat Commun..

[18] Madsen RR, Knox RG, Pearce W, Lopez S, Mahler-Araujo B, McGranahan N (2019). Oncogenic *PIK3CA* promotes cellular stemness in an allele dose-dependent manner. Proc Natl Acad Sci U S A..

[19] DePristo MA, Banks E, Poplin R, Garimella KV, Maguire JR, Hartl C (2011). A framework for variation discovery and genotyping using next-generation DNA sequencing data. Nat Genet..

[20] Pagès H. BSgenome: Software infrastructure for efficient representation of full genomes and their SNPs. R package. 2019.

[21] Carter SL, Cibulskis K, Helman E, McKenna A, Shen H, Zack T (2012). Absolute quantification of somatic DNA alterations in human cancer. Nat Biotechnol..

[22] Favero F, Joshi T, Marquard AM, Birkbak NJ, Krzystanek M, Li Q (2015). Sequenza: allele-specific copy number and mutation profiles from tumor sequencing data. Ann Oncol..

[23] Scheinin I, Sie D, Bengtsson H, van de Wiel MA, Olshen AB, van Thuijl HF (2014). DNA copy number analysis of fresh and formalin-fixed specimens by shallow whole-genome sequencing with identification and exclusion of problematic regions in the genome assembly. Genome Res..

[24] Nik-Zainal S, Davies H, Staaf J, Ramakrishna M, Glodzik D, Zou X (2016). Landscape of somatic mutations in 560 breast cancer whole-genome sequences. Nature..

[25] Zabransky DJ, Yankaskas CL, Cochran RL, Wong HY, Croessmann S, Chu D (2015). HER2 missense mutations have distinct effects on oncogenic signaling and migration. Proc Natl Acad Sci U S A..

[26] Yi KH, Lauring J (2016). Recurrent AKT mutations in human cancers: functional consequences and effects on drug sensitivity. Oncotarget..

[27] Leontiadou H, Galdadas I, Athanasiou C, Cournia Z (2018). Insights into the mechanism of the PIK3CA E545K activating mutation using MD simulations. Sci Rep..

[28] Baugh EH, Ke H, Levine AJ, Bonneau RA, Chan CS (2018). Why are there hotspot mutations in the TP53 gene in human cancers?. Cell Death & Differentiation..

[29] Ludyga N, Grünwald B, Azimzadeh O, Englert S, Höfler H, Tapio S (2012). Nucleic acids from long-term preserved FFPE tissues are suitable for downstream analyses. Virchows Arch..

[30] Boyden LM, Mao J, Belsky J, Mitzner L, Farhi A, Mitnick MA (2002). High bone density due to a mutation in LDL-receptor-related protein 5. N Engl J Med..

[31] Little RD, Carulli JP, Del Mastro RG, Dupuis J, Osborne M, Folz C (2002). A mutation in the LDL receptor-related protein 5 gene results in the autosomal dominant high-bone-mass trait. Am J Hum Genet..

[32] Bhat BM, Allen KM, Liu W, Graham J, Morales A, Anisowicz A (2007). Structure-based mutation analysis shows the importance of LRP5 beta-propeller 1 in modulating Dkk1-mediated inhibition of Wnt signaling. Gene..

[33] Nayar U, Cohen O, Kapstad C, Cuoco MS, Waks AG, Wander SA (2019). Acquired HER2 mutations in ER+ metastatic breast cancer confer resistance to estrogen receptor–directed therapies. Nature Genetics..

[34] Marusyk A, Almendro V, Polyak K (2012). Intra-tumour heterogeneity: a looking glass for cancer?. Nature Reviews Cancer..

[35] Seferbekova Z, Lomakin A, Yates LR, Gerstung M (2023). Spatial biology of cancer evolution. Nature Reviews Genetics..

[36] Kroigard AB, Larsen MJ, Laenkholm AV, Knoop AS, Jensen JD, Bak M (2018). Identification of metastasis driver genes by massive parallel sequencing of successive steps of breast cancer progression. PLoS One..

[37] Wang P, Bahreini A, Gyanchandani R, Lucas PC, Hartmaier RJ, Watters RJ (2016). Sensitive Detection of Mono- and Polyclonal ESR1 Mutations in Primary Tumors, Metastatic Lesions, and Cell-Free DNA of Breast Cancer Patients. Clin Cancer Res..

[38] Cheng DT, Mitchell TN, Zehir A, Shah RH, Benayed R, Syed A (2015). Memorial Sloan Kettering-Integrated Mutation Profiling of Actionable Cancer Targets (MSK-IMPACT): A Hybridization Capture-Based Next-Generation Sequencing Clinical Assay for Solid Tumor Molecular Oncology. J Mol Diagn..

[39] Ciriello G, Gatza ML, Beck AH, Wilkerson MD, Rhie SK, Pastore A (2015). Comprehensive Molecular Portraits of Invasive Lobular Breast Cancer. Cell..

[40] Jones S, Chen WD, Parmigiani G, Diehl F, Beerenwinkel N, Antal T (2008). Comparative lesion sequencing provides insights into tumor evolution. Proc Natl Acad Sci U S A..

[41] Bozic I, Antal T, Ohtsuki H, Carter H, Kim D, Chen S (2010). Accumulation of driver and passenger mutations during tumor progression. Proc Natl Acad Sci U S A..

[42] Nik-Zainal S, Alexandrov Ludmil B, Wedge David C, Van Loo P, Greenman Christopher D, Raine K (2012). Mutational Processes Molding the Genomes of 21 Breast Cancers. Cell..

