# Supplementary material for: Rare subclonal sequencing of breast cancers indicates putative metastatic driver mutations are predominately acquired after dissemination
Source: Genome Med. 2024 Feb 6;16:26. doi: 10.1186/s13073-024-01293-9 (PMC10848417; doi:10.1186/s13073-024-01293-9)
Supplement: Supplementary file 1 — Additional file 1. Contains the sections Supplemental Methods, Supplemental Results, and Supplemental Figures. [file 13073_2024_1293_MOESM1_ESM.pdf]

Supplemental Methods

Metastasis-specific mutations by WES

WES employed in (1) was used to identify mutations that were “metastasis-specific”, which are defined as those that were detected in metastases but not their corresponding primary tumors. Ensemble variant calling (MuTect1 v1.1.4 (2), MuTect2 - GATK v3.6 (2), and VarScan v2.3.4) was used to determine somatic mutations. Three tiers of confidence were assigned to resulting variants – tier I: coverage ≥ 30 reads, alternative allele read coverage (AAC) ≥ 6, VAF ≥ 0.10; tier II: coverage ≥ 10 reads, AAC ≥ 3, VAF ≥ 0.10; tier III: reported by at least two variant callers but did not meet tier I or II criteria. Mutations were determined to be metastasis-specific if they were tier I in the metastatic tumor and not tier I or tier II in the corresponding paired primary tumor. Depth of coverage and putative variants were determined within germline samples using samtools “mpileup” (v1.3.1, parameters: -t AD -Q 10) and bcftools “call” (v1.2, parameters: -AmO v) on WES of corresponding buffy coat blood samples.

Ultra-deep sequencing with unique molecular indices

smCounter outputs were used to determine resulting coverage at sequenced sites: “summary.xlsx” was used for average number of reads and unique molecular tags (MTs) per regions-of-interest (ROI) and “mtDepths.bedgraph” was used for site-specific MT coverage. For panel #1, ROIs were sequenced to an average coverage of 32,484x (median across samples, IQR = 6,175 - 40,441x) in primary tumors and 984x (IQR = 521 - 1,798x) in metastatic tumors. From these sequenced reads, the average unique molecular tag (MT) coverage was 1,283x (IQR = 792 - 2,503x) for primary tumors and 406x (IQR = 242 – 553x) for metastases. Panel #2 prioritized metastasis-specific mutations and was used to assay a subset of primary tumor blocks assayed in

panel #1 (11 tumor blocks from 10 primary tumors). For panel #2, ROIs were sequenced to average coverage of 133,731x (IQR = 97,100 – 202,547x), resulting in average MT coverage per ROI of 1,314x (IQR = 1006 – 3,207x).

Thirteen paired metastases were also assayed to determine concordance between sequencing technologies and to resolve clonality estimates of assayed mutations. Sequencing of the 11 remaining paired metastases was not possible due to tissue and extracted DNA being exhausted in our previous study (1). Eleven of the 13 assayed metastatic tumor blocks were fresh frozen. Average MT coverage in metastases was ~400x.

Sites within ROIs that were covered by at least 100 MTs by UDS-UMI and at least 8 reads by WES were considered adequately covered. Variants were limited to those that occurred at adequately covered sites, that passed all default filters by smCounter, had AAC ≥ 4 by UDS-UMI, and had AAC = 0 within the corresponding germline WES sample.

Nucleotide context of rare subclonal mutations

To determine the biological relevance of mutations called by UDS-UMI, tri-nucleotide contexts of mutations within the genome were determined using the BSgenome package (3) in R. An example of the syntax used is 5'-TCG, where C is the mutated reference allele, T is the nucleotide at the 5' or -1 position, and G is the nucleotide at the 3' or +1 position. The reverse complement of the context is reported when the reference allele of the mutation is either G or A.

Cancer cell fraction and detection power

A Bayesian approach was used to estimate the proportion of tumor cells in which a given mutation was present (cancer cell fraction, CCF) based on total read coverage, AAC, tumor

cellularity, locus-specific CN, and estimated number of mutated alleles (a.k.a multiplicity factor, *s*) for that mutation (4). Tumor cellularity, ploidy, and the estimated ratio of the major to minor alleles were estimated from corresponding WES samples of tumor samples by Sequenza (5). Shallow whole genome sequencing employed in (1) was segmented using QDNAseq (6) to determine CN, which was normalized by estimated tumor ploidy and cellularity.

Calculating the CCF using a binomial distribution dependent on the number of reads (WES) or MTs (UDS-UMI) covering the alternative allele and the aforementioned locus-specific parameters permits the uncertainty of the mutation CCF estimate to be modeled. The CCF with the highest likelihood ("maximum *a posteriori*", CCF) is provided throughout. The multiplicity factor required for this approach cannot be measured and is often heuristically chosen. Some studies set this parameter for every mutation to 1 (*i.e.*, only one allele is expected to contain the mutation) or to the largest possible value (the CN of the major allele), which lead to CCF estimates that are biased towards either higher values or zero, respectively. In this analysis, as a compromise, the multiplicity factor was set - unless otherwise stated - to the median of all possible multiplicity factors from 1 to major CN, which was estimated by Sequenza, such that:

$$s = max\left(\frac{median(\ 1\ to\ majorCN\ )}{majorCN\ +\ minorCN} \times relative\ CN\ \times ploidy, 1\right)$$

$$CCF_{MAP}\ = max_{ccf}\ Binomial(x =\ AAC, n\ =\ Coverage, \theta = p)$$

$$p = \frac{(cellularity\ \times ccf\ \times s)}{(1 - cellularity) \times 2\ +\ cellularity \times ploidy \times relative\ CN}$$

The smallest CCF for which there was ≥95% power to call a mutation with high-confidence, $minCCF_{95\%}$, was estimated for both WES and UDS-UMI to determine an upper-

bound on the clonality of a theoretically undetected mutation. For example, for an undetected mutation, cells harboring this mutation were either not present in the region of the tumor that was assayed or were present in a sufficiently small number of tumor cells to have not been detected by sequencing. Accordingly, $minCCF_{95\%}$ provides a measure for how large a clone must be in order to reliably detect a mutation residing within it. $minCCF_{95\%}$ was calculated by identifying the smallest CCF for which the cumulative distribution function of the above equation was ≥95%, where the number of theoretical alternative reads required for a mutation to be called by WES was 10% of the total coverage at that locus (min = 6). The number of theoretical alternative reads required for UDS-UMI was dependent on the total number of MTs sequenced and was conservatively estimated as a stepwise function based on the minimum AAC of high-confidence mutations called by smCounter: when MTs < 453, AAC = 4; MTs < 753, AAC = 5; MTs < 1,196, AAC = 6; MTs < 2,090, AAC = 7; MTs < 2,820, AAC = 8; MTs ≥ 2,820, AAC = 9.

Rare subclonal mutation detection probabilities

The probabilities that one, or two, metastasis-specific mutation(s) were called in corresponding primary tumors by chance were estimated using either the background rare subclonal mutation burden (RMB) that was estimated panel-wide or RMB that was estimated for each trinucleotide context. Per-sample background RMB was calculated by dividing the number of detected rare subclonal mutations (inflated by 1 if none were detected) by the number of (non-metastasis-specific) assayed sites. Context-specific background RMB was calculated by dividing the number of rare subclonal mutations detected at each context by the number of (non-metastasis-specific) sites for which that context was assayed. For trinucleotide contexts that did not exhibit mutations, the probability that a mutation was detected by chance at any one of those

contexts was set to 1 divided by the number of sites across all trinucleotide contexts that did not exhibit at least one mutation.

The non-context-specific probabilities that at least one, or at least two, metastasis-specific mutation(s) were called in corresponding primary tumor by chance were estimated using a binomial test where p = background RMB and n = the number of metastasis-specific mutations assayed in that patient. The context-specific probability of detecting at least one, or at least two, metastasis-specific mutations in a given primary tumor can be estimated by summing over the products of individual context-specific detection probabilities per assayed mutation over all possible combinations of successes, whereby at least one or at least two mutations were detected. In practice, this was implemented via simulation whereby each success was determined via comparison to a number pulled uniformly between 0 and 1 (1 million simulations). Similarly, the combined probabilities that at least one metastasis-specific mutation was detected by chance in six patients, and at least two metastasis-specific mutations were detected by chance in three patients, can be estimated by taking the sum of the products of individual detection probabilities over all possible combinations of detected patients (using context-specific and non-context-specific per-sample detection probabilities separately). This was also implemented via simulation.

To investigate the possibility that detected metastasis-specific mutations were acquired in in both primary and metastatic tumors independently (and thus were not linked by inheritance), we reasoned that we should not be able to detect, to the same extent, metastasis-specific mutations in primary tumors from which the metastasis did not arise. Permutation p-values were generated by finding the number of times at least one metastasis-specific mutation was detected in at least six permuted (*i.e.*, non-related) primary tumors and the number of times at least two

metastasis-specific mutations were detected in at least three permuted primary tumors from 10,000 permutations of sample labels.

Results of these tests are included in Table 1.

Estimating modes of dissemination

Modes of dissemination can be inferred from the CCF distribution of mutations shared between primary and metastatic tumors. Subclonal probability, $P_{CCF < 1}$, is determined by taking the sum of the probabilities for CCF values less than 1, which is intuitively the proportion of CCF posterior distribution that is less than 1. As utilized here, a mutation is likely to be subclonal if $P_{CCF < 1} \geq 0.95$. Alternatively, a mutation can be considered clonal if the CCF is close to 1 and the subclonal probability is not significant. A mutation that is likely subclonal in both primary and metastatic tumors in at least one tumor block suggests that the metastasis resulted from more than one subclone from the primary tumor (polyclonal dissemination). The presence of a subclonal mutation in the primary tumor and a clonal mutation in the metastatic tumor is indicative of monoclonal dissemination, in which all disseminated cells harbor the mutation. Note that this does not rule out the presence of additional subclones that are heterogeneous for other mutations.

Two mutations can be estimated via the pigeonhole principle to be within the same subclone if, for every pairwise combination of multiplicity factors, the sum of their CCF distributions is significantly greater than one. In this case, the CCF sum was determined by constructing a normal distribution with the mean being the sum of means of the separate CCF distributions and variance being the sum of variances of the separate CCF values. If 95% of the resulting normal distribution is greater than one, the mutations likely co-occurred within the

same subclone. In several cases, ambiguity in multiplicity factors precluded the ability to estimate polyclonal vs. monoclonal distribution, or whether mutations co-occurred within subclones due to mutations being equally like to occur in subclonal or clonal populations.

Supplemental Results

Mitigation of FFPE artifacts in UDS-UMI

To assess the efficacy of UDG treatment in mitigating FFPE artifacts, five primary tumor samples were sequenced twice, each with and without UDG treatment. Within rare subclones (VAF < 0.10), C>T mutations, which can result from FFPE-induced cytosine deamination, were estimated to be depleted by UDG treatment by at least 88% panel-wide; by at least 98% (median) at 5’-C[A/T/C] sites (range = 92.8 – 98.4%); and by at least 33% (median) at 5’-CG sites (range = 22.4 – 45.9%)(Fig. 1C and 1D). Whereas UDG is effective at removing uracil bases resulting from FFPE-induced cytosine deamination, the decrease in effectiveness observed at 5’-CG sites is likely due the inability of UDG treatment to remove artifacts that result from the deamination of 5-methylcytosine (5mC) to thymine, which occurs at 5’-CG (or CpG) sites.

However, C>T mutations at 5’-CG sites can also arise from the passive process of 5mC deamination by hydrolysis, an endogenous mutational process that is ubiquitous and prevalent across cancer types (referred to as “Signature 1” in the literature), correlated with patient age at diagnosis (7), and enriched in subclonal cellular populations (8). Furthermore, C>T mutations at 5’-TCG sites were 1.7-2.4X more frequent than C>T mutations at other 5’-CG sites, a pattern that is inconsistent with a relatively uniform distribution expected for C>T mutations arising from FFPE-induced 5mC deamination across 5’-CG sites. C>T mutations were also enriched at 5’-TCA sites (6% of mutations, 2% of sites), despite the ability of UDG to effectively ablate

FFPE artifacts at these sites. The non-uniform distribution of C>T mutations across 5’-CG sites, as well as the enrichment of C>T mutations at 5’-TCA sites, suggests that a substantial proportion of rare subclonal mutations identified did not arise from FFPE artifacts.

Furthermore, recurrent rare subclonal mutations within primary tumors were enriched with mutations in the COSMIC databases (one-sided Fisher’s exact test, $P = 0.0089$; 25.3% of recurrent versus 12.7% of non-recurrent mutations between primary tumors). For example, the *PIK3CA* H1047R mutation, a frequent driver of breast cancer (9), was found in rare subclones of two unrelated primary tumors. The enrichment for previously reported somatic mutations in cancer amongst mutations that were shared between unrelated tumor blocks, provide evidence that rare subclonal mutations are biologically relevant and do not primarily arise from sequencing noise and/or FFPE artifacts.

Evolutionary trajectories of metastatic recurrence

In patient P65, the *JAK1* G902V mutation was estimated to be present in a minor or rare subclone within the primary tumor (for $s = 1$, CCF = 0.37; for $s = 2$, CCF = 0.18) and clonal within its metastatic tumor to the liver (for $s = 1$, CCF = 1.94; for $s = 2$, CCF = 0.97). This suggests that all cells within this metastasis originated from a *JAK1*-mutant minor subclone present within the primary tumor (i.e., monoclonal dissemination), providing evidence that *JAK1* dysregulation may drive metastasis and/or treatment resistance.

In patient P39, two metastasis-specific mutations were identified within rare subclones in the primary tumor: *GHSR* (for $s = 1$, CCF = 0.060; for $s = 2$, CCF = 0.030; for $s = 3$, CCF = 0.020; for $s = 4$, CCF = 0.015) and *NPRL3* (for $s = 1$, CCF = 0.029; for $s = 2$, CCF = 0.015). Considering that a nonsense mutation in *CDKN1B* was found to be clonal within the metastasis

and was not detected within the primary tumor, this suggests that mutations within *GHSR* and *NPRL3* were likely present within the same rare subclone that was mono-clonally disseminated from the primary tumor. This conclusion is consistent with the mutation within *GHSR* being estimated to be clonal with the metastases, however, is not consistent within the estimated subclonality of the mutation in *NPRL3*. Thus, either the detection of the mutation in *NPRL3* within the primary tumor was likely a false positive, or the estimated clonality within the metastases by WES was erroneous.

For patient P26, the metastasis was estimated have originated from polyclonal dissemination based on the significant subclonality of the *CD14* mutation within one of the metastatic tumor blocks (Mt2). However, polyclonal dissemination is not consistent with the presence of a *TP53* mutation that was found to be clonal within this same metastatic tumor block (Mt2) and was not detected by UDS-UMI within the primary tumor, suggesting that this mutation arose after dissemination and swept throughout the tumor. Thus, either the clonality estimates for mutations in *TP53* or *CD14* with Mt2 were erroneous or the detection of the *CD14* mutation within the primary tumor was a false positive.

For patient P12, the metastasis was estimated to have originated from monoclonal dissemination based on the near clonal estimation of a mutation in *PIK3CA* that was clonal in the metastasis but not detected with the paired primary tumor. Both metastasis-specific mutations detected in the primary tumor P12 were specific to one of two primary tumor blocks assayed, pointing to the geographic specificity of this subclone within the primary tumor (as opposed to a dispersed population of cells). Furthermore, both mutations were estimated to be within 4.3% - 19% of cells, with ambiguity resulting from 4 possible multiplicity factors (i.e., number of alleles in which the mutation was present). Together, results suggest that the metastasis in this patient

resulted from monoclonal dissemination of a rare subclone that harbored both mutations and was present in 4-19% of cells in just 1 or 2 primary tumor regions sequenced.

Finally, for patient P11, mono - vs. polyclonal dissemination for the liver metastasis from P11 could not be determined confidently due to ambiguity regarding multiplicity factors. However, the presence and near clonal estimates (for s = 1) across 3 metastatic tumor blocks for both the *TGFBRAP1* mutation and the *XIRP2* mutation, of which the latter was not detected within the primary tumor by UDS-UMI, suggests that this metastasis resulted from monoclonal dissemination of a rare primary tumor subclone harboring *TGFBRAP1*.

A conceptual framework for the relative likelihoods of sequential mutation acquisition

For both driver and passenger models, the probability of a tumor of size (T) acquiring the first in a series of (k) mutations can be modeled as a Poisson process where λ = mutation rate (μ, which is defined by the number of mutations acquired on average per cell division) and T is the number of trials:

$$P_{sequence}(1) \propto 1 - P(failure\ to\ acquire\ a\ mutation)^T$$

$$P_{sequence}(1) \propto 1 - Poisson(X = 0, \lambda = \mu)^T$$

For a Poisson process, the probability of failure becomes roughly equal to $1 - \lambda$ with low values of λ. Therefore, considering that mutation rates in cancer cells have been estimated to be low (10^{-6} - 10^{-3} mutations per cell division)(10), we can say that the probability of acquiring at least one mutation within a tumor is:

$$P_{sequence}(1) \propto 1 - (1 - \mu)^T$$

Some amount of clonal expansion (c, fraction of cells) of this 1st-mutant cell could then occur due either to increased fitness conferred by the mutation or randomly via neutral evolution.

The resulting number of cells of the expanded 1st-mutant subclone thus determines the number of trials used to determine the probability of the acquiring a new mutation, whereby, in expectation:

$$\#\ of\ mutant\ cells = T\ \times\ c_1 \times\ P_{expansion}$$

Thus, the probability that a second mutation is acquired within this 1st-mutant subclone is a function of the mutation rate (μ) as well as the number of trials, which is determined by the relative size of the 1st-mutant subclone (c_1), the total tumor size (T), and the probability that the 1st-mutant subclone underwent clonal expansion ($P_{expansion}$).

$$P_{sequence}(2) \propto \left(1 -\ (1-\mu)^{(T\ \times\ c_1\ \times\ P_{expansion})}\right)\ \times\ P_{sequence}(1)$$

From this, the probability of sequentially acquiring a series of k mutations within the same clonal lineage can be estimated recursively and is proportional to:

$$P_{sequence}(k) \propto (1 -\ (1-\mu)^{(T\ \times\ c_{k-1}\ \times\ P_{expansion})})\ \times P_{sequence}(k-1)$$

$$P_{sequence}(k) \propto \prod_{1}^{k-1}\left(1 -\ (1-\mu)^{(T\ \times\ c_i\ \times\ P_{expansion})}\right) \times (1 -\ (1-\mu)^{T})$$

Assuming constant T, μ, c, and $P_{expansion}$, this can be reduced to:

$$P_{sequence}(k) \propto\ (1 -\ (1-\mu)^{(T\ \times\ c\ \times\ P_{expansion})})^{k-1} \times (1 -\ (1-\mu)^{T})$$

The above considerations indicate that the probability of a tumor subclone acquiring a series of mutations exponentially decreases with lower mutation rates, greater numbers of mutations, lower tumor sizes, lower extents of clonal expansion, and lower probabilities of clonal expansion. This general framework was then used to evaluate the likelihoods of driver versus passenger models of sequential acquisition on a relative basis.

The probability that a mutant-subclone becomes expanded ($P_{expansion}$) is drastically different between the passenger and driver models. To this end, driver mutations confer greater levels of fitness. Therefore, for driver mutations, $P_{expansion}$ is 1 because a subclone bearing the

$k + 1$ mutations has greater fitness than all other subclones that have at most k mutations (and corresponding levels of fitness conferred by the k mutations). In contrast, passenger mutations do not confer increased fitness. Accordingly, for passenger mutations, $P_{expansion}$ is inversely proportional to the total number of cells in the tumor ($1/T$) times the number of cells that can be feasibly expanded based on extents of clonal expansion ($1/c$).

$$driver\ mutation\ P_{expansion} = 1$$

$$passenger\ mutation\ P_{expansion} = \frac{1}{Tc}$$

From these probabilities of expansion, the following formulas can be used to estimate the probabilities of a acquiring a sequence of driver mutations or acquiring a sequence of passenger mutations.

$$P_{driver}(k) \propto \ (1 - \ (1 - \mu)^{Tc})^{k-1} \times (1 - \ (1 - \mu)^{T})$$

and

$$P_{passenger}(k) \propto \left(1 - \ (1 - \mu)^{\left(Tc \times \frac{1}{Tc}\right)}\right)^{k-1} \times (1 - \ (1 - \mu)^{T})$$

$$P_{passenger}(k) \propto \ \big(1 - (1 - \ \mu)\big)^{k-1} \ \times (1 - \ (1 - \mu)^{T})$$

$$P_{passenger}(k) \propto \ \mu^{k-1} \times (1 - \ (1 - \mu)^{T})$$

Thus, the probability of acquiring a series of driver mutations is far higher than the probability of acquiring a series of passenger mutations because for each mutation in the series there is a large pool of cells within which mutations can potentially be acquired (via a Poisson process where the number of trials is the number of cells). In contrast, acquiring a series of passenger mutations is far less likely because the number of cells in a potentially expanded subclone is offset in its entirety by the random probability of expansion. Mathematically, this probability becomes equivalent to the product of the initial probability of at least one cell

acquiring a mutation and the probability of acquiring the remaining $k-1$ passenger mutations in that same cell in the absence of clonal expansion (μ^{k-1}). Therefore, the relatively likelihood of the passenger model becomes exponentially less likely as mutation rate decreases or k increases.

Notably, this model can be extended to estimate the probability of sequentially acquiring (d) driver mutations in a series of k mutations, which can be expressed as

$$P_{sequence}(k,d) \propto P_{driver}(d) \times P_{passenger}(k-d)$$

$$P_{sequence}(k,d) \propto (1-(1-\mu)^{Tc})^{d} \times \mu^{k-d-1} \times (1-(1-\mu)^{T})$$

Thus, depending on the number of driver mutations, acquiring a mixture of driver and passenger mutations may also be exceedingly more likely than acquiring a series of exclusively passenger mutations.

From the principles outlined above, it is evident – especially at larger k – that the likelihood of acquiring a series of driver mutations is far higher than the likelihood of acquiring a series of passenger mutations ($P_{\text{passenger}} << P_{\text{driver}}$) within the same clonal lineage, because the former probability is offset by non-random and significant clonal expansion, thereby providing a larger population of potential target cells for each round of mutation.

Into the above probabilistic framework, we incorporated the number of sequentially arising metastasis-specific mutations that were identified ($k = 60, 8, and$ 3.4)(see Results). For the passenger model, the extent of clonal expansion (c) is irrelevant because it is canceled out in the model by the random probability of expansion (see above). For the driver model, (c) was set to the mean CCF of the identified mutations ($c = 0.79, 0.80, and$ 0.83). For estimation, we chose a hypothetical tumor with 10^6 cells and a mutation rate of $10^{-4.5}$, which was midpoint of the range that has been reported in cells (10).

Supplemental Figures

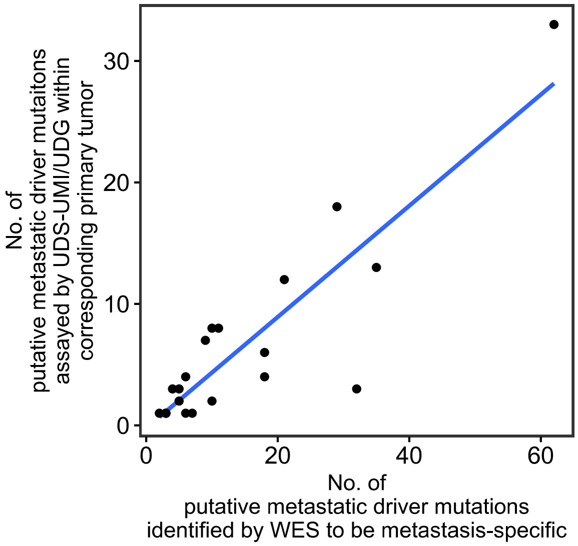

Fig. S1: The number of assayed putative metastatic driver mutations was proportional to the number of identified putative metastatic driver mutations. Dot plot showing the relationship between the number of total putative metastatic driver mutations that were identified in each patient by WES and were assayed within corresponding primary tumors by UDS-UMI/UDG.

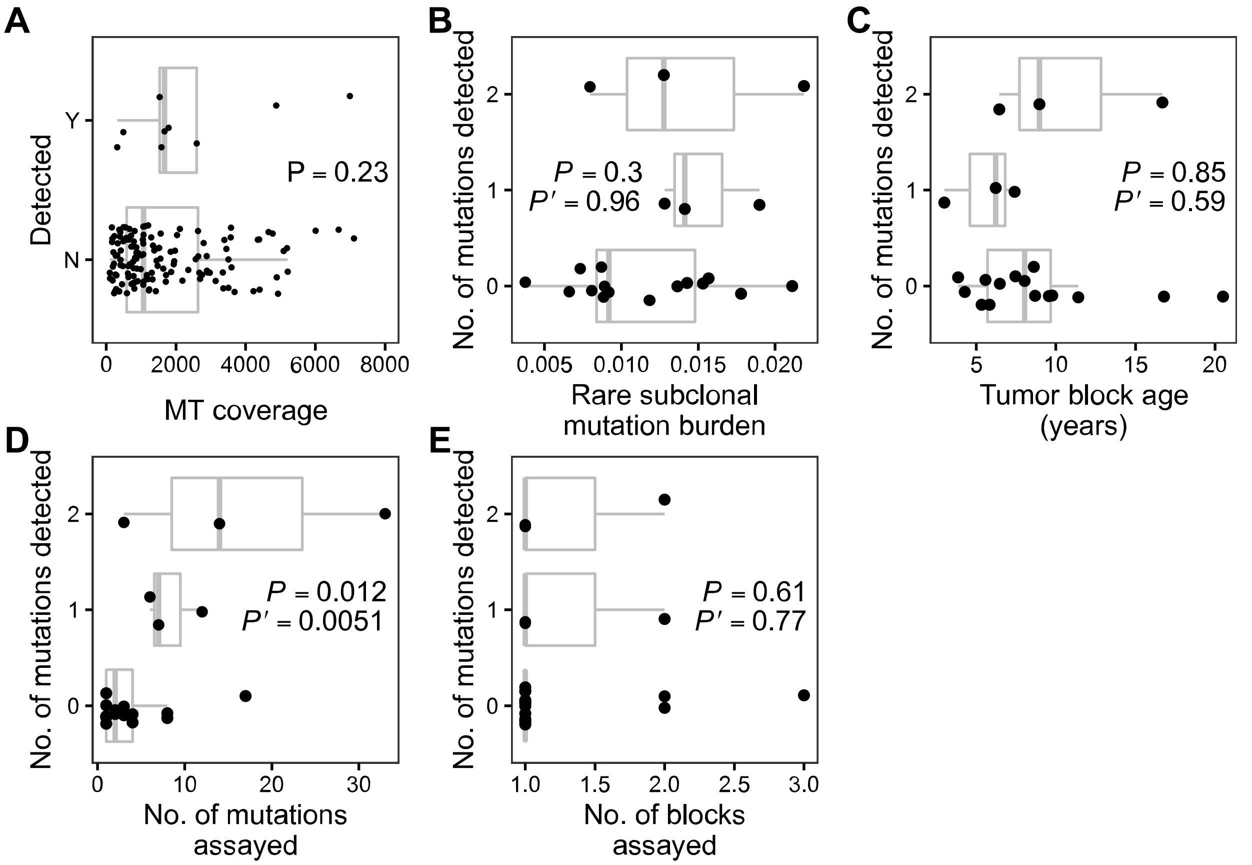

Fig. S2: Analysis of potential technical covariates of the ability to detect metastatic driver mutations within rare primary tumor subclones. (A) MT coverage as a function of mutations detection, indicating that no significant difference in MT coverage was observed for mutations that were detected versus those that were not. (B-E) Multivariate linear regression analysis of five potential technical covariates of the ability to detect metastatic driver mutations within patients. *P*-values indicate significance from univariate Kendall tau tests, *P'*-values indicate significance from a multivariate test that included all five potential covariates. (B and C) Rare subclonal mutation burden within each primary tumor and tumor block age of each primary were not correlated the number of metastatic driver mutations detected in each patient, suggesting that detected mutations did not result from high background mutation rates that were potentially induced by FFPE artifacts related to tumor block age. (D) More metastatic driver mutations were likely to be detected when more mutations were assayed. (E and F) Number of tumor blocks assayed per patient and average MT coverage were not correlated with the number of metastatic driver mutations detected in each patient.

Patient	# of Metastatic Driver Mutations Assayed	# of Metastatic Driver Mutations Detected	Probability of Detecting by Chance ≥1 Mutation (context specific)	Probability of Detecting by Chance ≥2 Mutations (context specific)
P25	3	2	**0.024 (0.082)**	**0.00019 (0.00022)**
P39	14	2	0.27 (0.71)	**0.037 (0.26)**
P12	33	2	0.48 (0.78)	0.14 (0.42)
P11	6	1	**0.075 (0.0035)**	
P65	7	1	0.13 (0.28)	
P26	12	1	0.16 (0.38)	
combined likelihood			**0.018 (0.023)**	**0.0012 (0.015)**
permutation			**0.029**	**0.022**

Table S1: Probability of erroneously detecting metastatic driver mutation within rare primary tumor subclones. For each patient, probabilities that the number of metastatic driver mutations detected within rare primary tumor subclones resulted from the detection of false positives was calculated as a function of the number of mutations assayed and the background frequency of mutations detected within rare subclones at non-mutation sites. Probabilities that utilized mutational frequencies specific to trinucleotide sites are given in parentheses. Combined likelihoods across patients and sample label permutation analyses indicate that it was unlikely that at least one mutation was detected by chance in at least six patients, and that at least two mutations were detected by chance in at least three patients.

References

1. Paul MR, Pan TC, Pant DK, Shih NN, Chen Y, Harvey KL, et al. Genomic landscape of metastatic breast cancer identifies preferentially dysregulated pathways and targets. *J Clin Invest.* 2020.
2. DePristo MA, Banks E, Poplin R, Garimella KV, Maguire JR, Hartl C, et al. A framework for variation discovery and genotyping using next-generation DNA sequencing data. *Nat Genet.* 2011;43(5):491-8.
3. Pagès H. BSgenome: Software infrastructure for efficient representation of full genomes and their SNPs. R package. 2019.
4. Carter SL, Cibulskis K, Helman E, McKenna A, Shen H, Zack T, et al. Absolute quantification of somatic DNA alterations in human cancer. *Nat Biotechnol.* 2012;30(5):413-21.
5. Favero F, Joshi T, Marquard AM, Birkbak NJ, Krzystanek M, Li Q, et al. Sequenza: allele-specific copy number and mutation profiles from tumor sequencing data. *Annals of Oncology.* 2015;26(1):64-70.
6. Scheinin I, Sie D, Bengtsson H, van de Wiel MA, Olshen AB, van Thuijl HF, et al. DNA copy number analysis of fresh and formalin-fixed specimens by shallow whole-genome sequencing with identification and exclusion of problematic regions in the genome assembly. *Genome Res.* 2014;24(12):2022-32.
7. Alexandrov LB, Nik-Zainal S, Wedge DC, Aparicio SAJR, Behjati S, Biankin AV, et al. Signatures of mutational processes in human cancer. *Nature.* 2013;500(7463):415-21.
8. Letouzé E, Shinde J, Renault V, Couchy G, Blanc JF, Tubacher E, et al. Mutational signatures reveal the dynamic interplay of risk factors and cellular processes during liver tumorigenesis. *Nat Commun.* 2017;8(1):1315.
9. Madsen RR, Knox RG, Pearce W, Lopez S, Mahler-Araujo B, McGranahan N, et al. Oncogenic PIK3CA promotes cellular stemness in an allele dose-dependent manner. *Proc Natl Acad Sci U S A.* 2019;116(17):8380-9.
10. Duesberg P, Stindl R, and Hehlmann R. Explaining the high mutation rates of cancer cells to drug and multidrug resistance by chromosome reassortments that are catalyzed by aneuploidy. *Proc Natl Acad Sci U S A.* 2000;97(26):14295-300.
